# BRAF and MEK Inhibitors Influence the Function of Reprogrammed T Cells: Consequences for Adoptive T-Cell Therapy

**DOI:** 10.3390/ijms19010289

**Published:** 2018-01-18

**Authors:** Jan Dörrie, Lek Babalija, Stefanie Hoyer, Kerstin F. Gerer, Gerold Schuler, Lucie Heinzerling, Niels Schaft

**Affiliations:** 1 Department of Dermatology, Universitätsklinikum Erlangen and Faculty of Medicine, Friedrich-Alexander-Universität Erlangen-Nürnberg (FAU), 91052 Erlangen, Germany; jan.doerrie@uk-erlangen.de (J.D.); Babalija@students.uni-marburg.de (L.B.); stefanie.hoyer@uk-erlangen.de (S.H.); Kerstin.Gerer@web.de (K.F.G.); gerold.schuler@uk-erlangen.de (G.S.); lucie.heinzerling@uk-erlangen.de (L.H.); 2 Department of Genetics, Friedrich-Alexander-Universität Erlangen-Nürnberg, 91058 Erlangen, Germany

**Keywords:** BRAF inhibitor, MEK inhibitor, kinase inhibitor, CAR-T cell, dabrafenib, trametinib, vemurafenib, cobimetinib, melanoma, immunotherapy

## Abstract

BRAF and MEK inhibitors (BRAFi/MEKi), the standard treatment for patients with BRAF^V600^ mutated melanoma, are currently explored in combination with various immunotherapies, notably checkpoint inhibitors and adoptive transfer of receptor-transfected T cells. Since two BRAFi/MEKi combinations with similar efficacy are approved, potential differences in their effects on immune cells would enable a rational choice for triple therapies. Therefore, we characterized the influence of the clinically approved BRAFi/MEKi combinations dabrafenib (Dabra) and trametinib (Tram) vs. vemurafenib (Vem) and cobimetinib (Cobi) on the activation and functionality of chimeric antigen receptor (CAR)-transfected T cells. We co-cultured CAR-transfected CD8^+^ T cells and target cells with clinically relevant concentrations of the inhibitors and determined the antigen-induced cytokine secretion. All BRAFi/MEKi reduced this release as single agents, with Dabra having the mildest inhibitory effect, and Dabra + Tram having a clearly milder inhibitory effect than Vem + Cobi. A similar picture was observed for the upregulation of the activation markers CD25 and CD69 on CAR-transfected T cells after antigen-specific stimulation. Most importantly, the cytolytic capacity of the CAR-T cells was significantly inhibited by Cobi and Vem + Cobi, whereas the other kinase inhibitors showed no effect. Therefore, the combination Dabra + Tram would be more suitable for combining with T-cell-based immunotherapy than Vem + Cobi.

## 1. Introduction

The RAS/RAF/MEK/ERK MAPK pathway is a key signaling pathway involved in the regulation of normal cell proliferation, survival, and differentiation [1]. Under normal circumstances, the serine/threonine kinase BRAF is activated by NRAS (neuroblastoma RAS viral oncogene homolog) [2,3] and in turn phosphorylates the downstream proteins MEK1/2 (MAP2K, mitogen-activated protein kinase kinase), which then activate ERK1/2 [4]. Activated ERKs translocate to the nucleus, where they phosphorylate and regulate different transcription factors, which leads to changes in gene expression [5]. However, common oncogenic mutational activation of NRAS or BRAF is observed in human tumors [6,7]. Approximately 50–60% of metastatic melanomas contain an activating mutation in the BRAF oncogene. Of the mutations in BRAF, over 90% affect amino acid position 600, with the vast majority resulting in substitution of a valine into a glutamic acid (BRAF^V600E^), but also other substitutions at this position are found [6]. These genetic alterations result in constitutive activation of the MAPK signaling pathway, which supports cell proliferation and tumor cell growth through several mechanisms, including reduced apoptosis, increased metastatic potential, invasiveness, and immune suppression [6,8,9,10]. As a consequence of this knowledge, new therapeutic approaches using specific inhibitors as targeted therapy against the MAPK signaling pathway members were developed for the treatment of melanoma patients.

BRAF kinase inhibitors (BRAFi) like vemurafenib (Vem; marketed as Zelboraf) and dabrafenib (Dabra) have become the standard targeted therapy for melanoma patients with BRAF mutations [11,12]. Unfortunately, after the first evidence of objective response, most patients developed resistance to BRAFi monotherapies which was manifested by progressive disease and rapid relapse often caused by a reactivation of the MAPK pathway, e.g., appearance of additional NRAS or other MEK-activating mutations [13,14].

To address this problem, specific MEK inhibitors (MEKi) were developed to additionally inhibit the cascade further downstream. Combined treatment with Vem and the MEKi cobimetinib (Cobi) or Dabra and the MEKi trametinib (Tram) resulted in an increase of progression-free survival, compared to BRAFi alone [15,16]. Both these combinations of BRAFi/MEKi were recently approved by both the FDA and the European Commission for the treatment of advanced melanoma patients. Nevertheless, secondary resistance develops frequently [15,16]. Therefore, several phases II and III trials are currently evaluating triple therapies of BRAF/MEK inhibitor therapy plus checkpoint inhibitor treatment with anti-PD1 (NCT02910700, NCT02967692, NCT02858921, NCT02130466). Other immunological treatment modalities are also being investigated as combination partners.

As a new therapeutic approach to potentially increase survival and delay relapse, these clinically applied BRAFi/MEKi could be combined with cellular immunotherapy, e.g., chimeric antigen receptor (CAR)-T-cell therapy.

Because the MAPK pathway is also involved in immune cell function and survival, BRAFi and MEKi are likely to influence immune functions. Previous studies have shown that BRAF and MEK inhibitors may have an influence on immune cells and can modulate their functions [17,18,19]. The effects of the BRAFi must be explained with a lack of specificity for the mutated BRAF because the non-malignant cells only harbor the wild-type version of this kinase. The MEKi, in contrast, target non-mutated MEK1/2, and could thus possibly interfere with a pathway essential for the activity of immune cells. MEK-inhibitors as monotherapy to treat melanoma patients with MAPK pathway activating mutations other than BRAF^V600^ are currently being explored [20].

Several CARs against different antigens also expressed on melanoma were already tested in clinical trials (NCT03060356, NCT01218867, NCT02107963, NCT02830724). In previous work [21], we have generated a CAR specific for chondroitin sulfate proteoglycan 4 (CSPG4), also known as melanoma-associated chondroitin sulfate proteoglycan (MCSP), or high molecular weight melanoma-associated antigen (HMW-MAA), which is a cell-surface antigen expressed on 90% of melanoma primary tumors and metastases, but also on sarcomas, astrocytomas, gliomas, and neuroblastomas [22,23,24,25], and therefore we consider this an ideal target antigen. We have shown that T cells transfected with this CAR mediated effective antigen-specific tumor cell lysis in vitro and in vivo and also induced the secretion of pro-inflammatory cytokines [21].

A combination of BRAFi/MEKi treatment with CSPG4-specific CAR-T-cell therapy would be a new and probably more efficient approach for melanoma therapy. To analyze the possible immunological effects of BRAFi/MEKi, we tested in vitro how the application of these kinase inhibitors influences the functionality of CAR-transfected T cells. We studied in detail CAR-T-cell activation, cytokine secretion, and cytolytic capacity, and found differential effects of the two different BRAFi/MEKi combinations on these CAR-T-cell functions.

The findings of this study are highly relevant for the future use of BRAF and MEK inhibitors in combination with adoptive CAR-T-cell therapy or other immunotherapies. Of the two approved BRAFi/MEKi combinations, the Dabra + Tram combination had a much smaller negative effect on CAR-T-cell functionality than the Vem + Cobi combination. Our data provide a clear rationale for the combination of targeted therapy and immunotherapy for melanoma and may further expand the understanding of BRAF and MEK inhibitor effects on the immune system.

## 2. Results

### 2.1. Antigen-Specific Activation of CAR-T Cells Is Differentially Affected by BRAFi/MEKi Treatment

To study the effects of BRAF and MEK inhibitor (BRAFi/MEKi) treatment on antigen-specific activation of chimeric antigen receptor (CAR)-T cells, we added these inhibitors to co-incubations at concentrations similar to serum levels detected in patients (Table 1) [16,26,27].

**Figure 1:**
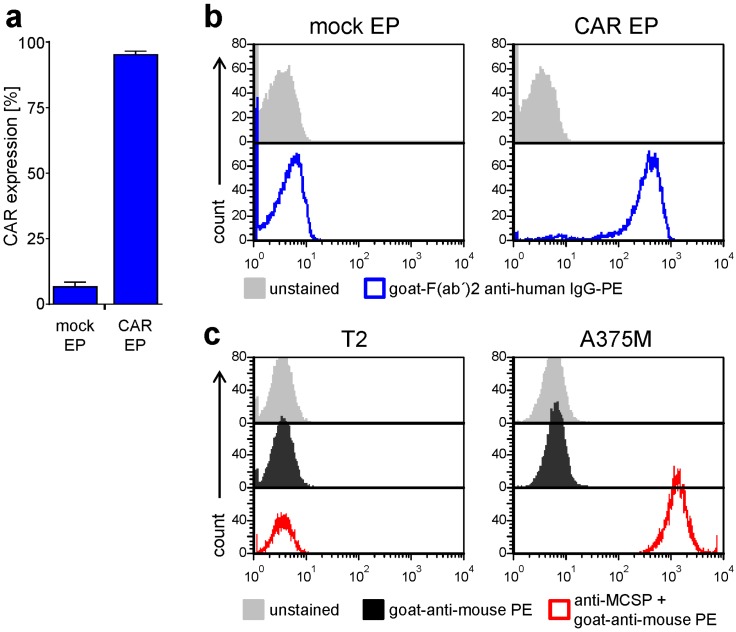
CSPG4-specific CAR and CSPG4 expression on effector T cells and target cells, respectively. (**a**,**b**) Peripheral blood mononuclear cells (PBMCs) were isolated from the blood of healthy donors through density gradient centrifugation and CD8^+^ T cells were isolated with magnetic activated cell sorting (MACS) beads. These cells were either mock electroporated (EP) or were transfected with RNA encoding the CSPG4-specific CAR. CAR expression was flow-cytometrically determined 4 h after electroporation by staining with a PE-labeled goat-F(ab′)2 anti-human IgG antibody. (**a**) Percentage of CAR-positive cells (average of 8 independent experiments + SEM; original data see Table S1) and (**b**) Histograms of one typical experiment (grey histograms: unstained cells, and blue histograms: goat-F(ab′)2 anti-human IgG-PE stained cells) are shown. (**c**) The CSPG4 antigen expression on the target cell lines T2 and A375M was determined by flow cytometry after primary staining with a CSPG4-specific antibody and secondary staining PE-labeled with goat-anti-mouse. Grey histograms: unstained cells; black histograms: secondary only staining; red histograms: primary anti-CSPG4 staining + secondary staining.

CD8^+^ T cells isolated from blood of healthy donors were electroporated either without RNA (mock), or with RNA encoding the CSPG4-specific CAR [21]. After RNA electroporation, approximately 95% of the CD8^+^ T cells expressed the CSPG4-specific CAR (Figure 1a,b). Four hours after electroporation these CAR-T cells were co-incubated with the CSPG-negative cell line T2 and the CSPG4^+^ melanoma cell line A375M at a 1:1 ratio in the absence or presence of the different kinase inhibitors. To check for CSPG4 expression on the target cells, staining of CSPG4 was carried out on the T2 and the A375M cell lines (Figure 1c). A high CSPG4 expression was observed on the A375M cell line, while the T2 cell line was negative for CSPG4 (Figure 1c).

**Figure 2:**
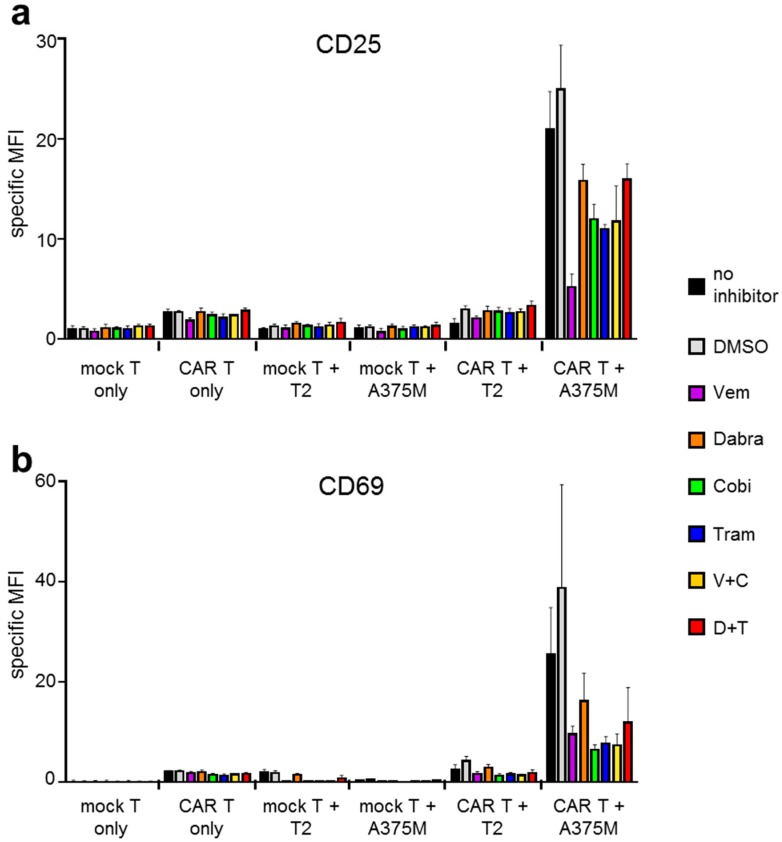
BRAF and MEK inhibitor treatment affects CAR-T-cell activation after antigen-specific stimulation. CAR-T cells were generated as described in Figure 1. Four hours after electroporation, these cells were co-incubated overnight with CSPG4-negative T2 cells and the CSPG4^+^ melanoma cell line A375M at a 1:1 ratio. Mock-transfected T cells were used as control. Co-incubations were performed in the absence of inhibitors (no inhibitor), in the presence of DMSO only (solvent control), or in the presence of the different kinase inhibitors, either alone or in combination. The used kinase inhibitors vemurafenib (Vem, V), dabrafenib (Dabra, D), cobimetinib (Cobi, C), and trametinib (Tram, T) were used in final concentrations listed in Table 1. Mock-transfected T cells (mock T) stimulated with T2 or A375M, and mock-transfected T cells and CAR-T cells incubated without target cells served as negative controls. After 16 h of co-incubation, the cells were harvested and stained for the activation markers CD25 (**a**) and CD69 (**b**) and measured by flow cytometry. The specific mean fluorescence intensity (MFI) of the respective activation markers on cells in the T-cell gate is depicted. The MFI was calculated by subtracting the value of the respective isotype control. Data are presented as mean + SEM derived from four independent experiments (original data see Table S2; for statistical analyses see Tables S3 and S4). Flow-cytometric data of a representative donor is shown in Supplemental Figure S1.

To measure T-cell activation, CD25 and CD69 expression on the CAR-T cells were determined after overnight incubation of effector T cells with target cells. Incubations of T cells only (i.e., without target cells) resulted in a minor upregulation of CD25 and CD69 expression on CAR-transfected T cells compared to mock-transfected T cells (Figure 2), which was independent of the presence or absence of inhibitors. This upregulation is probably caused by the intrinsic activity of the signaling modules (i.e., CD28 and CD3ζ) contained in the CAR. Similar levels of CD25 and CD69 upregulation were seen after co-incubation with the CSPG-negative target T2 (Figure 2). Mock-transfected T cells did not upregulate CD25 or CD69 expression after co-incubation with T2 or A375M target cells (Figure 2).

Importantly, co-incubation with the CSPG4^+^ target A375M resulted in a differential upregulation of CD25 and CD69 expression depending on the inhibitor present during the co-incubation (Figure 2). For CD25 expression, the antigen-specific upregulation was highest without inhibitor and with DMSO (solvent control) (Figure 2a). The presence of Vem significantly decreased the CD25 upregulation after antigen-specific stimulation (Figure 2a). Incubation with the MEK inhibitors Tram and Cobi alone, but also the combination of Vem + Cobi reduced the CD25 upregulation approximately to 50% (Figure 2a). Dabra alone had a significantly weaker effect on CD25-upregulation than Vem alone, and the combination Dabra + Tram resulted in the mildest effects on CD25 upregulation (Figure 2a). For CD69 expression a similar trend was seen, except that incubation with Vem alone, Tram alone, Cobi alone, but also the combination of Vem + Cobi resulted in a comparably low CD69 expression (Figure 2b). Again, stimulation in the presence of Dabra alone and Dabra + Tram resulted in the mildest effects on CD69 expression (Figure 2b).

In summary, these data indicate that BRAFi and MEKi can reduce antigen-specific T-cell activation and that this influence depends on the type of BRAF and MEK inhibitor.

### 2.2. Antigen-Specific Cytokine Secretion by CAR-T Cells Is Differentially Affected by BRAFi/MEKi Treatment

**Figure 3:**
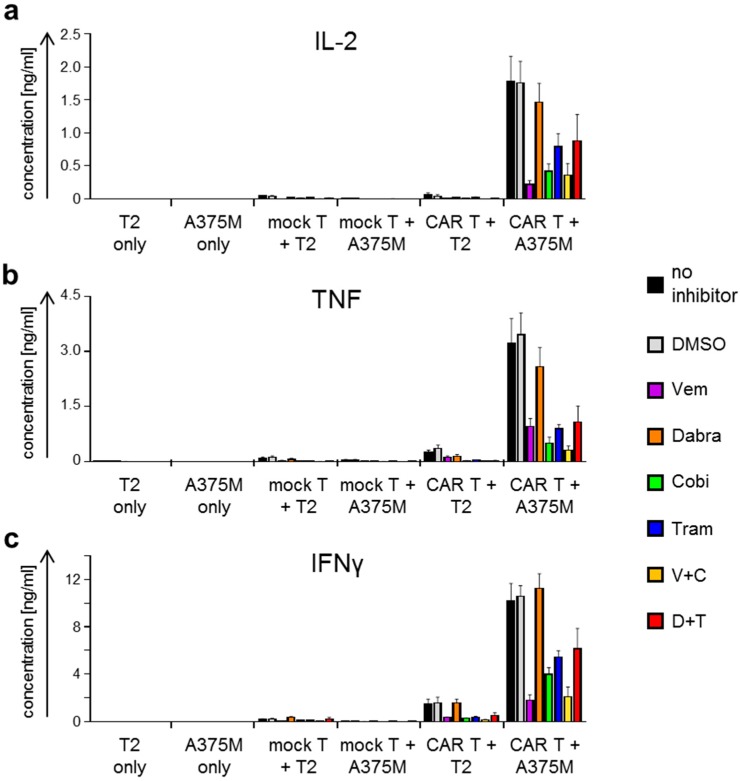
BRAF and MEK inhibitor treatment affects CAR-T-cell cytokine secretion after antigen-specific stimulation. CAR-T cells were generated as described in Figure 1. Four hours after electroporation, these cells were co-incubated overnight with CSPG4-negative T2 cells and the CSPG4^+^ melanoma cell line A375M at a 1:1 ratio. Mock-transfected T cells (mock T) were used as control. Co-incubations were performed in the absence of inhibitors (no inhibitor), in the presence of DMSO only (solvent control), or in the presence of the different kinase inhibitors, either alone or in combination. The used kinase inhibitors vemurafenib (Vem, V), dabrafenib (Dabra, D), cobimetinib (Cobi, C), and trametinib (Tram, T) were added in final concentrations listed in Table 1. T2 and A375M cells without T cells and mock-transfected T cells stimulated with T2 or A375M served as negative controls. Concentrations of interleukin (IL)-2 (**a**), tumor necrosis factor (TNF) (**b**), and interferon gamma (IFNγ) (**c**) were determined after overnight co-incubation with a Cytometric Bead Array (CBA), and are depicted in [ng/mL]. Data are presented as mean + SEM of four independent experiments (original data, see Table S5; for statistical analyses see Tables S6–S8).

**Figure 4:**
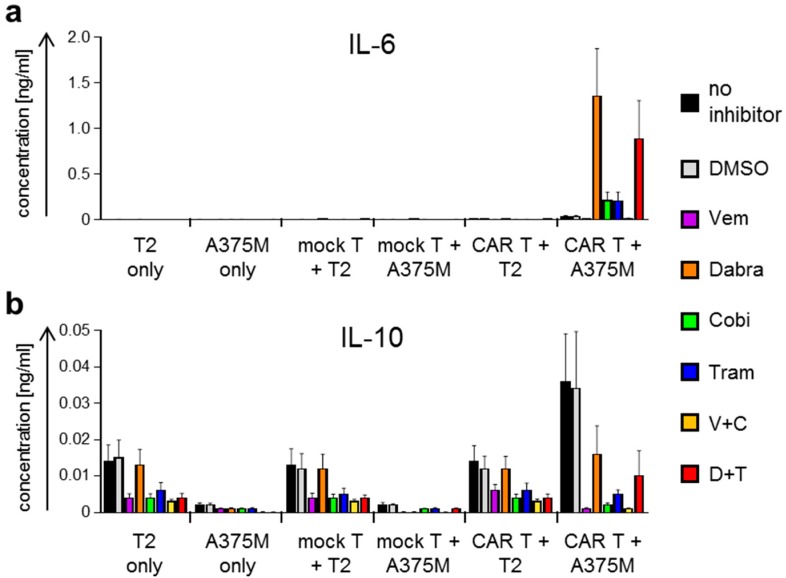
BRAF and MEK inhibitor treatment affects CAR-T-cell cytokine secretion after antigen-specific stimulation. CAR-T cells were generated as described in Figure 1. Four hours after electroporation, these cells were co-incubated overnight with CSPG4-negative T2 cells and the CSPG4^+^ melanoma cell line A375M at a 1:1 ratio. Mock-transfected T cells (mock T) were used as control. Co-incubations were performed in the absence of inhibitors (no inhibitor), in the presence of DMSO only (solvent control), or in the presence of the different kinase inhibitors, either alone or in combination. The used kinase inhibitors vemurafenib (Vem, V), dabrafenib (Dabra, D), cobimetinib (Cobi, C), and trametinib (Tram, T) were added in final concentrations listed in Table 1. T2 and A375M cells without T cells and mock-transfected T cells stimulated with T2 or A375M served as negative controls. Concentrations of IL-6 (**a**) and IL-10 (**b**) were determined after overnight co-incubation with a CBA, and are depicted in [ng/mL]. Data are presented as mean + SEM of four independent experiments (original data, see Table S9; for statistical analyses see Tables S10 and S11).

Since cytokine secretion by CAR-T cells after antigen-specific stimulation corresponds to immune activation, we investigated the differential effect of the kinase inhibitors. To do so, we generated T-cell populations as described above and incubated these overnight with T2 and A375M target cells at a 1:1 ratio in the absence or presence of BRAF and MEK inhibitors (as indicated; Figure 3 and Figure 4). Supernatants were collected and cytokine content was determined using a Cytometric Bead Array (CBA) detecting interleukin (IL)-2, IL-4, IL-6, IL-10, tumor necrosis factor (TNF), and interferon gamma (IFNγ) (Figure 3 and Figure 4).

Co-incubations of CAR-T cells with A375M target cells led to an antigen-specific secretion of the pro-inflammatory cytokines IL-2, TNF, and IFNγ (Figure 3). For all these cytokines, a similar pattern was observed: high secretion in the conditions without inhibitor and DMSO (solvent control) (Figure 3). For the bona-fide CTL-cytokine IFNγ, the different inhibitors displayed specific inhibitory effects: only Dabra alone had no effect, while Vem alone had the strongest inhibitory effect (Figure 3c). Both MEKi significantly reduced IFNγ-secretion but the effect of Cobi alone was significantly stronger (Figure 3c). The condition with Vem + Cobi was similarly inhibited as Vem alone, while the Dabra + Tram condition was significantly less inhibited (Figure 3c). The cytokines IL-2 and TNF behaved similarly, however, they did not reach significance in some of the comparisons (Figure 3a,b). CAR-T cells incubated with T2 cells secreted detectable but clearly lower quantities of these cytokines, pointing, like CD25 and CD69 expression in these conditions, toward an intrinsic activity of the CAR. CAR-T cells incubated in the absence of target cells produced cytokine amounts similar to or lower than those incubated with T2 cells (data not shown).

Interestingly, IL-6 secretion behaved differently. Substantial quantities of this cytokine were only produced upon stimulation in the presence of Dabra alone, Dabra + Tram, and to a much lower extent in the presence of Tram alone or Cobi alone. In all other conditions IL-6 secretion was absent (Figure 4a). Low quantities of IL-10 were secreted by T2 cells independent of the presence of T cells (Figure 4b). The presence of Vem alone, Tram alone, Cobi alone, Vem + Cobi, and Dabra + Tram, but not of Dabra alone seemed to reduce these quantities to approximately 50% (Figure 4b). No secretion of IL-4 was observed (data not shown).

Taken together, the results clearly show that BRAFi and MEKi differentially influenced antigen-specific cytokine secretion by CAR-T cells. It is of note that of the two clinically used combinations of BRAFi/MEKi, Dabra + Tram had a much smaller negative effect on CAR-induced cytokine secretion than Vem + Cobi.

### 2.3. BRAFi/MEKi Treatment Differentially Affects Antigen-Specific Lytic Capacity of CAR-Transfected T Cells

**Figure 5:**
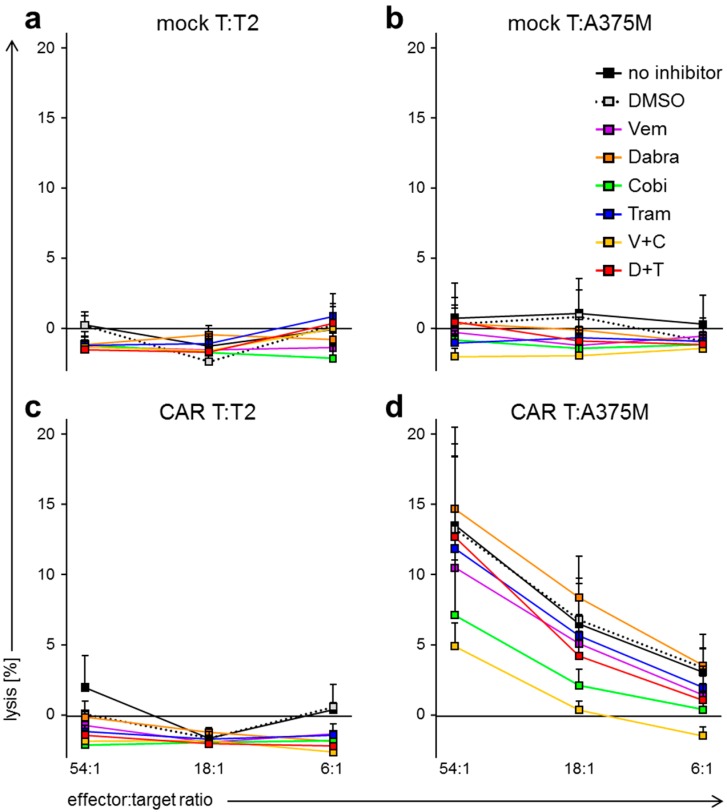
BRAF and MEK inhibitor treatment affects CAR-T-cell lytic capacity after antigen-specific stimulation. T cells were generated as described in Figure 1, and electroporated without RNA (mock T; (**a**,**c**)) or with CAR-RNA (CAR T; (**b**,**d**)). Twenty-four hours after electroporation, the cytolytic capacity of these cells toward the CSPG4-negative T2 cells (**a**,**d**) and the CSPG4^+^ melanoma cell line A375M (**b**,**d**) was examined at indicated effector to target ratios in a standard 4 to 6 h chromium release assay. Co-incubations were performed in the absence of inhibitors (no inhibitor), in the presence of DMSO only (solvent control), or in the presence of the different kinase inhibitors, either alone or in combination. The used kinase inhibitors vemurafenib (Vem, V), dabrafenib (Dabra, D), cobimetinib (Cobi, C), and trametinib (Tram, T) were supplemented in final concentrations listed in Table 1. The release of chromium into the supernatant was determined and lysis was calculated as described in materials and methods. Data are presented as mean + SEM derived from three independent experiments, each performed in technical triplicates (for original data see Table S12; for statistical analyses see Table S13).

To assess the effects of BRAFi/MEKi treatment on the most important task of CAR-transfected T cells in adoptive immunotherapy, we determined whether BRAFi/MEKi treatment affects the antigen-specific lytic capacity. T cell populations were generated as described above to be used in a standard 4–6 h Cr^51^-release assay at different effector to target ratios (as indicated; Figure 5), with T2 and A375M as target cells. The Cr^51^-release assays were performed in the absence or presence of BRAFi/MEKi.

Mock-transfected T cells did not induce lysis of T2 or A375M target cells, independent of the inhibitor condition (Figure 5a,b). Furthermore, CAR-T cells did not lyse T2 target cells (Figure 5c). Considering the lysis of the melanoma cell line A375M, we observed a similar lytic capacity of the CAR-T cells in the conditions without inhibitor, with DMSO solvent control, with Vem alone, Tram alone, Dabra alone, and the combination Dabra + Tram (Figure 5d). In contrast, the condition containing Cobi alone showed significantly reduced lytic capacity of the CAR-T cells, and this lytic capacity was even further reduced in the additional presence of Vem (Vem + Cobi; Figure 5d).

As already described above for cytokine secretion, these experiments showed the differential effect of the BRAFi and MEKi on antigen-specific lytic capacity of CAR-T cells. Cobi alone already reduced the lytic capacity. The combination Dabra + Tram had a significantly weaker negative impact on the lytic capacity than Vem + Cobi.

## 3. Discussion

The strategy of combining BRAFi and MEKi with immunotherapy requires a better understanding of the effects of kinase inhibition on normal immune cell function. Although the two currently approved combinations of BRAFi and MEKi appear similarly effective against melanoma [28], their effects on healthy cells, not bearing BRAF mutations, but employing the respective signaling pathway, may significantly differ. BRAFi have for example a paradoxical effect on wild-type BRAF [29], which is more pronounced for Vem than for Dabra [30]. The specificity of the MEKi for MEK1 and MEK2 also varies [31]. Therefore, the different BRAFi and MEKi may differentially interfere with the various effector functions of CAR-transfected T cells. Tumor rejection depends on T-cell activation and subsequent cytokine secretion and lytic activity of these T cells. Thus, in this study, we thoroughly investigated the effects of these inhibitors on activation, cytokine secretion, and the cytolytic capacity of CSPG4-specific CAR-transfected CD8^+^ T-cell in in vitro assays.

In both the antigen-specific activation of CAR-T cells and the antigen-specific cytokine secretion by CAR-T cells, we observed the mainly inhibitory effects of the BRAFi and MEKi used, which would argue against a combination with CAR-T-cell-based immunotherapy. However, the different inhibitors and their combinations clearly varied in the intensity of these effects: Vem alone, Cobi alone, Tram alone, and Vem + Cobi had the largest negative influence, while Dabra alone had the mildest negative influence. Of note is that of the two clinically used combinations of BRAFi/MEKi, Dabra + Tram had a much smaller negative effect than Vem + Cobi. This was also the case when looking at the lytic capacity of CAR-T cells.

Considering antigen-specific cytokine secretion by CAR-T cells, several observations are important for an intended combined clinical application of BRAFi/MEKi with CAR-T cells, since efficacy as well as toxicity can be influenced:

(i) The pro-inflammatory cytokines IL-2, TNF, and IFNγ are important for a good T-cell response against the tumor. IL-2 promotes the differentiation of T cells into effector and memory T cells [32], TNF was originally described as anti-tumorigenic [33], and IFNγ has a number of important functions including macrophage activation, major histocompatibility complex induction, and Th1 differentiation [34]. However, the downside of an efficient secretion of these cytokines can be a type of systemic inflammatory immune response, which is similar to severe infections and characterized by symptoms like hypotension, pyrexia, tachycardia, headache, swelling, redness, or nausea [35]. This so-called cytokine release syndrome (CRS) is a feared side effect of CAR-T-cell therapy caused by a massive systemic release of pro-inflammatory cytokines by the transferred cells [36,37,38,39]. In the serum of patients where CRS was observed, pro-inflammatory cytokines like IL-6, TNFα, and IFNγ were consistently elevated [40]. CAR-transfected T cells were least compromised in the production of these cytokines in the presence of Dabra alone compared to the other kinase inhibitors. Secretion of these cytokines was reduced by the presence of Dabra + Tram compared to Dabra alone. This might have a positive effect on the reduction of CRS side effects. Since IL-2, TNF, and IFNγ are nevertheless necessary for an anti-tumor response, the use of Dabra + Tram might form a good balance between preventing an exaggerated cytokine release causing CRS on the one hand and a minimum secretion of the cytokines seen in the setting with Vem + Cobi on the other hand.

(ii) Dabrafenib facilitated the CAR-induced secretion of a very high quantity of IL-6, whereas T cells stimulated in the absence of kinase inhibitors did not produce this cytokine. In the presence of the MEKi some IL-6 was also produced antigen-specifically. In the presence of Vem, no IL-6 was detected. Interestingly, some studies have shown that constitutive activation of the MAPK pathway by the BRAF^V600E^ mutation induces the downstream production of IL-6 [10,41]. The release of IL-6 we observed was probably not caused by a paradoxical effect of Dabra, because it would then also be expected with Vem. Therefore, the molecular reasons for this observation remain to be elucidated. IL-6 is a multifunctional cytokine that plays a central role in host defense due to its wide range of immune and hematopoietic activities and its potent ability to induce the acute phase response [42]. On the other hand, IL-6 plays a central role in CRS [40]. Due to the fact that CRS is a severe and potentially deadly side effect, the application of Dabra should only be combined with CAR-T-cell therapy together with Tram, because this mitigates the IL-6 secretion and thus also the possible side effects of CRS.

(iii) CAR-transfected T cells did not secrete any IL-4 and secreted only very low quantities of IL-10, which were further reduced by the different BRAFi and MEKi. Since IL-10 is an anti-inflammatory cytokine that supports melanoma cell proliferation and inhibits anti-tumor responses, and as production of IL-4 can induce IL-10 secretion [43], the production of these anti-inflammatory cytokines after application of CAR-T cells in the patient could lead to an inhibition of tumor-reactive T cells and prevent effective recognition and lysis of cancer cells and even promote melanoma growth and should therefore be avoided.

Others have also tested the effect of therapeutically relevant inhibitor concentrations on the cytolytic capacity of CAR-T cells [26]. In contrast to our results, Gargett et al. [26] showed that Vem alone clearly inhibited the cytolytic capacity of these CAR-T cells, and Dabra combined with Tram also inhibited the cytolytic capacity, but to a lesser extent. In line with our results, Dabra alone did not inhibit the lytic activity. It is important to note in this case that the concentrations of Dabra and Tram were chosen at the higher end of the patient plasma range [26]. Furthermore, Gargett et al. [26] incubated the CAR-T cells for 48 h in the presence of BRAFi/MEKi without stimulation, and then these cells were co-cultured for 6 h in the presence of these kinase inhibitors with chromium-labeled target cell lines. Moreover, they tested T cells from melanoma patients, whereas we tested T cells from healthy donors. Finally, the observed differences might be explained by the use of a third generation CAR containing CD3ζ, CD28, and OX-40 signaling domains by Gargett and co-workers [26].

Our findings not only have consequences for the use of BRAFi/MEKi in combination with CAR-T cells, but also in a more general sense considering immune responses or combinations with other immunotherapies. For example, our finding that the MEK inhibitor Cobi used as a single agent can inhibit the lytic capacity of T cells might be of importance in studies using MEKi without BRAFi in the setting of melanoma with non-mutated BRAF but mutated NRAS [20]. Anti-tumor T-cell responses could be influenced in such settings. Moreover, other combinations of MAPK-pathway-targeted therapy and immunotherapy were tested for melanoma as well. For example, it was shown that the combination of checkpoint inhibitors with BRAFi and MEKi is reasonable but dangerous and can cause severe side effects [44]. A combination of Dabra, Tram, and ipilimumab was tested, resulting in colitis followed by intestinal perforation in two out of seven patients [44], which was caused by Tram, since in the combination of Dabra and ipilimumab, only one grade 3 colitis was observed in 25 patients. It is not clear yet how Tram, or any MEK inhibitor, contributes to the toxicity of ipilimumab [44]. Other authors tested in a phase I study a combination of a PD-L1-antibody with Dabra and Tram in metastatic melanoma patients with mutated BRAF^V600^ [45], and showed that a combination is possible.

Although we have investigated the influence of the BRAFi and MEKi on immunotherapeutical effector functions of the CAR-T cells, the differential effects on the intracellular signaling induced by the CAR remain to be elucidated. The next steps to understand the observed differences should be a thorough analysis of the phosphorylation state of the respective signaling cascade upon CAR stimulation in the presence of the inhibitors. In this context, also the influence on the mechanism of killing should be addressed to distinguish between granzyme/perforin- and death-receptor-mediated killing. Such knowledge will be valuable in understanding the different effects of the different inhibitors, and help in the improvement of such therapies and the design of new small molecule inhibitors of the RAS/RAF/MEK/ERK-pathway.

## 4. Materials and Methods

### 4.1. Cell Culture Media

R10 medium is RPMI 1640 (Lonza, Basel, Switzerland) supplemented with final concentrations of 10% (*v*/*v*) heat-inactivated fetal bovine serum (PAA, GE Healthcare, Piscataway, NY, USA), 2 mM l-glutamine (Lonza), 100 U/mL penicillin, 100 μg/mL streptomycin (Lonza), 2 mM HEPES (PAA, GE Healthcare), and 20 μM β-mercaptoethanol (Gibco, Life Technologies, Carlsbad, CA, USA).

### 4.2. BRAF and MEK Inhibitors

All inhibitors were purchased as pure substances: vemurafenib (PLX4032) from Adooq Bioscience, Irvine, CA, USA, cobimetinib (GDC-0973), trametinib (GSK1120212), and dabrafenib (GSK2118436A) from AbMole BioScience, Houston, TX, USA. BRAF and MEK inhibitor concentrations used in our in vitro experiments were based on the description on the package insert of the providers and published serum concentrations [16,26,27]. Used final concentrations are summarized in Table 1.

### 4.3. Cell Lines

T2 (ATCC^®^ CRL-1992™) is a CSPG-negative TAP-deficient TxB hybrid cell line. The CSPG4^+^ A375M melanoma cell line (ATCC^®^ CRL-3223™) was described previously by Kozlowski et al. [46]. Both cell lines were cultured in R10 medium.

### 4.4. T-Cell Isolation

All human material from healthy volunteers was obtained after written informed consent for inclusion before they participated in the study. The study was conducted in accordance with the Declaration of Helsinki, and the protocol was approved by the institutional review board of the Friedrich-Alexander-Universität Erlangen-Nürnberg (date: 14 September 2016; reference number: 251_16 B). Peripheral blood mononuclear cells (PBMCs) were purified by density centrifugation using the Lymphoprep reagent (Axis-Shield poC AS, Oslo, Norway). CD8^+^ T cells were isolated by Magnetic Activated Cell Sorting (MACS) using CD8-specific microbeads (Miltenyi Biotech, Bergisch Gladbach, Germany). The CD8^+^ fraction was cultured in R10 medium supplemented with 10 ng/mL IL-7 at a concentration of 1 × 10^6^ cells/mL. The isolated cells were rested overnight at 37 °C until they were used for further experimental procedures.

### 4.5. RNA Transfection

The composition of the CSPG4-specific CAR was described previously [21] and featured a CD28-CD3ζ CAR backbone [47] 5′ of the IgG-spacer region. The DNA encoding the CARs was inserted into the pGEM4Z-5′UTR-sig-husurvivin-DC.LAMP-3′UTR RNA-production vector [48] (kindly provided by Kris Thielemans), replacing the sig-husurvivin-DC.LAMP sequence. RNA was produced using the mMESSAGE mMACHINE T7 Ultra kit (Life Technologies, Carlsbad, CA, USA). RNA was purified with an RNeasy Kit (Qiagen, Hilden, Germany). Electroporation of T cells was performed as described in detail previously [49].

### 4.6. Staining of CSPG4-Specific CAR on Transfected T Cells and CSPG4 on Target Cells

For the analysis of CSPG4-specific CAR on transfected T cells, these cells were stained 4 h after electroporation. Detection of CAR-expression was performed by using a goat F(ab’)2 anti-human IgG-RPE antibody (Southern Biotech, Birmingham, AL, USA, CSGP4-expression on the surface of target cell lines was determined using purified mouse-anti-CSPG4 antibody; clone 9.2.27 (BD). The secondary antibody used was PE-conjugated goat-anti-mouse-Ig polyclonal antibody (BD). Expression was measured directly via a FACScan cytometer (BD). Results were evaluated with CellQuest software (BD) and FCS Express software (FCS Express 5 Flow Research Edition) (DeNovo Software, Glendale, CA, USA).

### 4.7. Staining of T-Cell Activation Markers

T cells were used 4 h after electroporation, and were co-cultured with the target cells A375M or T2 overnight in R10 medium at a 1:1 ratio with 10^6^ cells per mL in total, in the absence or presence of BRAFi/MEKi. The activation markers CD25 and CD69 on T cells were analyzed by flow cytometry. T cells were stained with anti-CD25-FITC and CD69-PE antibodies and surface marker expression was measured directly via FACScan cytometer (BD). T cells were distinguished from target cells by forward/sideward scatter gating and results were evaluated with CellQuest software (BD) and FCS Express software (DeNovo Software). The specific mean fluorescence intensity (MFI) was calculated by subtraction of the background MFI obtained with isotype antibodies by using mouse IgG1 κ isotype control FITC antibody (BD) and mouse IgG1 isotype control PE antibody (Miltenyi Biotech), respectively.

### 4.8. Cytokine Secretion Assay

T cells were used 4 h after electroporation and were co-cultured with the target cells A375M or T2 overnight in R10 medium at a 1:1 ratio with 106 cells per mL in total, in the absence or presence of BRAFi/MEKi. The cytokine concentrations in the supernatants were determined with the Cytometric Bead Array human Th1/Th2 Cytokine Kit II (BD Bioscience, Heidelberg, Germany).

### 4.9. Chromium Release Assay

The cytolytic capacity of CAR-RNA-transfected T cells was determined in a standard chromium release assay [50]. Briefly, A375M and T2 cells were labeled with 100 µCi of Na_2_^51^CrO_4_/10^6^ cells. Target cells were washed and subsequently cultured in 96-well plates (Thermo Fisher, Waltham, MA, USA) at 1000 cells/well. The T cells were added at the indicated effector:target ratios in the absence or presence of different kinase inhibitors (as indicated), either alone or in combination. Cells were co-incubated in triplicate culture wells for 4–6 h. To determine spontaneous background release, target cells were incubated with R10 medium, whereas target cells cultured with 1% Nonidet-40 were used to determine maximum release. Radioactivity in the supernatant was determined and lysis was calculated as follows: ((measured release − background release)/(maximum release − background release) × 100%).

### 4.10. Figure Preparation and Statistical Analysis

Graphs were created and statistical analysis was performed using GraphPad Prism, Version 7 (GraphPad Software, La Jolla, CA, USA). *p*-Values were analyzed using a paired Students *t*-test. * indicates *p* ≤ 0.05, ** indicates *p* ≤ 0.01, and *** indicates *p* ≤ 0.001.

## 5. Conclusions

Taken together, this study shows that BRAFi/MEKi influence immune functions. Since these influences are highly dependent on the type of inhibitor, one has to carefully consider the differential effects in the choice of combination trials. Considering the data presented above, we suggest that CAR-T-cell therapy should be combined with Dabra + Tram rather than with Vem + Cobi. Our data provide relevant scientific evidence to support further investigation of a combination of Dabra + Tram and CAR-T cell therapy in clinical trials. 

## Figures and Tables

**Table 1:** Concentrations of kinase inhibitors used either alone or in combination in in vitro experiments (see materials and methods for more details).

Kinase Inhibitor	Target	Final Concentration
Vemurafenib (Vem, V) ^1^	BRAF^V600E^	60 µM
Dabrafenib (Dabra, D) ^2^	BRAF^V600^	1 µM
Trametinib (Tram, T) ^3^	MEK1/2	30 nM
Cobimetinib (Cobi, C) ^4^	MEK1/2	0.5 µM
V + C	BRAF^V600E^ + MEK1/2	60 µM + 0.5 µM
D + T	BRAF^V600^ + MEK1/2	1 µM + 30 nM

^1^ marketed under Zelobraf^®^ by Roche; ^2^ marketed under Tafinlar^®^ by Novartis; ^3^ marketed under Mekinist^®^ by Novartis; ^4^ marketed under Cotellic^®^ by Roche.

## Supplementary Materials

Supplementary materials can be found at www.mdpi.com/1422-0067/19/1/289/s1.

## Abbreviations

CAR	chimeric antigen receptor
CSPG4	chondroitin sulfate proteoglycan 4
BRAF	v-Raf murine sarcoma viral oncogene homolog B
MEK	Mitogen-activated protein kinase kinase
Dabra	Dabrafenib
Tram	Trametinib
Vem	Vemurafenib
Cobi	Cobimetinib
ERK	extracellular signal-regulated kinases
MAPK	Mitogen-activated protein kinase
NRAS	Neuroblastoma RAS viral oncogene homolog
BRAFi	BRAF kinase inhibitor
MEKi	MEK inhibitor
FDA	Food and Drug Administration
PD1	Programmed cell death protein 1
MCSP	Melanoma-associated chondroitin sulfate proteoglycan
HMW-MAA	high molecular weight-melanoma associated antigen
RNA	Ribonucleic acid
IL	Interleukin
TNF	Tumor necrosis factor
IFN	Interferon
DMSO	Dimethyl sulfoxide
CRS	Cytokine release syndrome
MACS	Magnetic-activated cell sorting
PBMC	peripheral blood mononuclear cell
