# Supplementary material for: BRAF and MEK Inhibitors Influence the Function of Reprogrammed T Cells: Consequences for Adoptive T-Cell Therapy
Source: Int J Mol Sci. 2018 Jan 18;19(1):289. doi: 10.3390/ijms19010289 (PMC5796234; doi:10.3390/ijms19010289)
Supplement: Supplementary file 1 [file ijms-19-00289-s001.pdf]

International Journal of
*Molecular Sciences*

MDPI

Supplementary Materials

# BRAF and MEK Inhibitors Influence the Function of Reprogrammed T Cells: Consequences for Adoptive T-Cell Therapy

**Jan Dörrie, Lek Babalija, Stefanie Hoyer, Kerstin F. Gerer, Gerold Schuler, Lucie Heinzerling and Niels Schaft**

**Table S1.** Original data used for Figure 1A [1].

| Donor | Percentage positive cells | |
|---|---|---|
| | mock electroporated | CAR-RNA electroporated |
| HD 1 | 8.79 | 96.59 |
| HD 2 | 2.9 | 86.75 |
| HD 3 | 4.59 | 97.53 |
| HD 4 | 11.33 | 97.91 |
| HD 5 | 6.76 | 96.84 |
| HD 6 | 2.16 | 93.85 |
| HD 7 | 15.91 | 98.29 |
| HD 8 | 1.08 | 93.79 |
| Average (n=8) | 6.69 | 95.19375 |
| SEM (n=8) | 1.80138835 | 1.35063204 |

[1] Percentage positive cells was determined by flow cytometry. See legend to figure 1 and materials and methods section for details.

**Table S2.** Original data used for Figure 2 [1].

| | HD 1 | | | | | |
|---|---|---|---|---|---|---|
| | CD25 (MFI) | | | | | |
| | **Mock T only** | **CAR-T only** | **Mock T + T2** | **Mock T + A375M** | **CAR T + T2** | **CAR T + A375M** |
| **no inhibitor** | 0.56 | 1.93 | 0.91 | 0.73 | 2.32 | 18.66 |
| **DMSO** | 0.55 | 2.49 | 1.44 | 0.75 | 3.81 | 17.53 |
| **Vem** | 0.4 | 1.8 | 0.56 | 0 | 2.26 | 3.95 |
| **Dabra** | 0.64 | 2.57 | 1.75 | 1.08 | 4.1 | 14.23 |
| **Cobi** | 0.66 | 2.05 | 1.23 | 0.57 | 3.77 | 16.33 |
| **Tram** | 0.55 | 2.2 | 1.31 | 0.75 | 3.53 | 11.42 |
| **V+C** | 0.92 | 2.49 | 1.52 | 0.84 | 3.55 | 20.87 |
| **D+T** | 1.18 | 2.73 | 1.84 | 0.86 | 4.42 | 17.02 |
| | CD69 (MFI) | | | | | |
| | **Mock T only** | **CAR-T only** | **Mock T + T2** | **Mock T + A375M** | **CAR T + T2** | **CAR T + A375M** |
| **no inhibitor** | 0 | 2.15 | 2.72 | 0.11 | 4.48 | 17.33 |
| **DMSO** | 0 | 2.83 | 2.38 | 0.11 | 5.96 | 12.83 |
| **Vem** | 0 | 1.95 | 0 | 0 | 1.8 | 6.1 |
| **Dabra** | 0 | 2.63 | 2.01 | 0.08 | 4.67 | 9.64 |
| **Cobi** | 0 | 1.48 | 0 | 0 | 1.84 | 9.37 |
| **Tram** | 0 | 1.63 | 0.14 | 0 | 2.33 | 5.54 |
| **V+C** | 0 | 1.64 | 0 | 0 | 1.23 | 6.08 |
| **D+T** | 0 | 1.59 | 0.05 | 0 | 1.62 | 4.95 |

| | HD 2 | | | | | |
|---|---|---|---|---|---|---|
| | CD25 (MFI) | | | | | |
| | **Mock T only** | **CAR-T only** | **Mock T + T2** | **Mock T + A375M** | **CAR T + T2** | **CAR T + A375M** |
| **no inhibitor** | 1.62 | 2.66 | 1.38 | 1.93 | 2.24 | 11.26 |
| **DMSO** | 1.3 | 2.76 | 1.33 | 1.7 | 2.1 | 17.04 |
| **Vem** | 1.2 | 2.43 | 1.77 | 1.43 | 2.37 | 8.05 |
| **Dabra** | 1.96 | 3.82 | 1.89 | 1.96 | 2.68 | 18.36 |
| **Cobi** | 1.38 | 2.82 | 1.67 | 1.79 | 3.07 | 11.74 |
| **Tram** | 1.55 | 2.85 | 2.02 | 1.7 | 3.19 | 12.06 |
| **V+C** | 1.41 | 2.36 | 1.81 | 1.49 | 2.04 | 5.01 |
| **D+T** | 1.52 | 3.35 | 2.41 | 2.02 | 3.79 | 15.64 |
| | CD69 (MFI) | | | | | |
| | **Mock T only** | **CAR-T only** | **Mock T + T2** | **Mock T + A375M** | **CAR T + T2** | **CAR T + A375M** |
| **no inhibitor** | 0 | 2.65 | 1.74 | 0.44 | 2.68 | 11.22 |
| **DMSO** | 0 | 2.75 | 1.65 | 0.27 | 2.22 | 16.36 |
| **Vem** | 0 | 1.84 | 0.19 | 0 | 1.71 | 8.8 |
| **Dabra** | 0.13 | 2.95 | 1.4 | 0.34 | 2.27 | 14 |
| **Cobi** | 0 | 2.46 | 0.03 | 0.06 | 1.61 | 6.44 |
| **Tram** | 0 | 2.27 | 0.25 | 0 | 1.83 | 7.79 |
| **V+C** | 0 | 1.61 | 0 | 0 | 1.27 | 3.68 |
| **D+T** | 0 | 2.17 | 0 | 0.07 | 1.97 | 5.49 |

| | **HD 3** | | | | | |
|---|---|---|---|---|---|---|
| | CD25 (MFI) | | | | | |
| | **Mock T only** | **CAR-T only** | **Mock T + T2** | **Mock T + A375M** | **CAR T + T2** | **CAR T + A375M** |
| **no inhibitor** | 0.01 | 3.13 | 0.69 | 0.32 | 1.41 | 27.14 |
| **DMSO** | 0.58 | 2.25 | 0.51 | 0.56 | 2.74 | 31.55 |
| **Vem** | 0.04 | 1.04 | 0.26 | 0.16 | 1.16 | 2.08 |
| **Dabra** | 0.38 | 1.7 | 0.77 | 0.48 | 1.78 | 18.7 |
| **Cobi** | 0.68 | 1.67 | 0.84 | 0.36 | 1.92 | 9.74 |
| **Tram** | 0.16 | 0.91 | 0.33 | 0.85 | 1.82 | 10.34 |
| **V+C** | 0.79 | 2.05 | 0.54 | 0.75 | 2.44 | 13.76 |
| **D+T** | 0.7 | 2.1 | 0.47 | 0.47 | 2.44 | 19.25 |
| | CD69 (MFI) | | | | | |
| | **Mock T only** | **CAR-T only** | **Mock T + T2** | **Mock T + A375M** | **CAR T + T2** | **CAR T + A375M** |
| **no inhibitor** | 0 | 2.47 | 2.84 | 0.15 | 2.98 | 53.1 |
| **DMSO** | 0 | 2.03 | 2.51 | 0.68 | 5.52 | 100.14 |
| **Vem** | 0 | 2.53 | 0.36 | 0.09 | 2.53 | 13.71 |
| **Dabra** | 0 | 1.21 | 1.6 | 0.34 | 2.8 | 32.6 |
| **Cobi** | 0 | 1.03 | 0.5 | -0.07 | 1.48 | 5.21 |
| **Tram** | 0 | 0.4 | 0.32 | 0.48 | 1.28 | 11.65 |
| **V+C** | 0 | 1.74 | 0.41 | 0.36 | 2.02 | 14.09 |
| **D+T** | 0 | 1.71 | 2.63 | 0.47 | 3.35 | 32.74 |

| | **HD 4** | | | | | |
|---|---|---|---|---|---|---|
| | CD25 (MFI) | | | | | |
| | **Mock T only** | **CAR-T only** | **Mock T + T2** | **Mock T + A375M** | **CAR T + T2** | **CAR T + A375M** |
| **no inhibitor** | 1.55 | 3.08 | 0.86 | 1.23 | 0 | 26.75 |
| **DMSO** | 1.45 | 3.08 | 1.66 | 1.49 | 3.18 | 33.58 |
| **Vem** | 1.23 | 1.98 | 1.48 | 1.15 | 2.31 | 6.57 |
| **Dabra** | 1.37 | 2.47 | 1.5 | 1.26 | 2.54 | 11.96 |
| **Cobi** | 1.33 | 2.97 | 1.4 | 1.02 | 2.17 | 9.9 |
| **Tram** | 1.54 | 2.42 | 1.02 | 1.41 | 1.92 | 10.01 |
| **V+C** | 1.77 | 2.54 | 1.6 | 1.45 | 2.64 | 7.28 |
| **D+T** | 1.69 | 3.03 | 1.83 | 1.85 | 2.52 | 11.72 |
| | CD69 (MFI) | | | | | |
| | **Mock T only** | **CAR-T only** | **Mock T + T2** | **Mock T + A375M** | **CAR T + T2** | **CAR T + A375M** |
| **no inhibitor** | 0 | 0.84 | 0.27 | 0.4 | 0 | 20.02 |
| **DMSO** | 0 | 1.19 | 0.66 | 0.69 | 3.08 | 25.37 |
| **Vem** | 0 | 0.9 | 0.14 | 0.37 | 0.4 | 9.62 |
| **Dabra** | 0 | 0.89 | 0.56 | 0.13 | 1.59 | 8.31 |
| **Cobi** | 0 | 0.7 | 0.01 | 0 | 0.08 | 4.42 |
| **Tram** | 0 | 0.55 | 0 | 0.15 | 0.72 | 5.71 |
| **V+C** | 0 | 1.06 | 0.08 | 0.1 | 0.95 | 5.36 |
| **D+T** | 0 | 0.98 | 0 | 0.48 | 0.13 | 4.32 |

| | **Average n=4** |
|---|---|
| | CD25 (MFI) |

| | Mock T only | CAR-T only | Mock T + T2 | Mock T + A375M | CAR T + T2 | CAR T + A375M |
|---|---|---|---|---|---|---|
| **no inhibitor** | 0.935 | 2.7 | 0.96 | 1.0525 | 1.4925 | 20.9525 |
| **DMSO** | 0.97 | 2.645 | 1.235 | 1.125 | 2.9575 | 24.925 |
| **Vem** | 0.7175 | 1.8125 | 1.0175 | 0.685 | 2.025 | 5.1625 |
| **Dabra** | 1.0875 | 2.64 | 1.4775 | 1.195 | 2.775 | 15.8125 |
| **Cobi** | 1.0125 | 2.3775 | 1.285 | 0.935 | 2.7325 | 11.9275 |
| **Tram** | 0.95 | 2.095 | 1.17 | 1.1775 | 2.615 | 10.9575 |
| **V+C** | 1.2225 | 2.36 | 1.3675 | 1.1325 | 2.6675 | 11.73 |
| **D+T** | 1.2725 | 2.8025 | 1.6375 | 1.3 | 3.2925 | 15.9075 |
| | CD69 (MFI) | | | | | |
| | **Mock T only** | **CAR-T only** | **Mock T + T2** | **Mock T + A375M** | **CAR T + T2** | **CAR T + A375M** |
| **no inhibitor** | 0 | 2.0275 | 1.8925 | 0.275 | 2.535 | 25.4175 |
| **DMSO** | 0 | 2.2 | 1.8 | 0.4375 | 4.195 | 38.675 |
| **Vem** | 0 | 1.805 | 0.1725 | 0.115 | 1.61 | 9.5575 |
| **Dabra** | 0.0325 | 1.92 | 1.3925 | 0.2225 | 2.8325 | 16.1375 |
| **Cobi** | 0 | 1.4175 | 0.135 | -0.0025 | 1.2525 | 6.36 |
| **Tram** | 0 | 1.2125 | 0.1775 | 0.1575 | 1.54 | 7.6725 |
| **V+C** | 0 | 1.5125 | 0.1225 | 0.115 | 1.3675 | 7.3025 |
| **D+T** | 0 | 1.6125 | 0.67 | 0.255 | 1.7675 | 11.875 |

| | **SEM n=4** | | | | | |
|---|---|---|---|---|---|---|
| | CD25 (MFI) | | | | | |
| | **Mock T only** | **CAR-T only** | **Mock T + T2** | **Mock T + A375M** | **CAR T + T2** | **CAR T + A375M** |
| **no inhibitor** | 0.39 | 0.28 | 0.15 | 0.35 | 0.54 | 3.78 |
| **DMSO** | 0.24 | 0.18 | 0.25 | 0.28 | 0.36 | 4.43 |
| **Vem** | 0.30 | 0.29 | 0.36 | 0.36 | 0.29 | 1.33 |
| **Dabra** | 0.36 | 0.44 | 0.25 | 0.30 | 0.48 | 1.64 |
| **Cobi** | 0.20 | 0.31 | 0.17 | 0.32 | 0.42 | 1.54 |
| **Tram** | 0.35 | 0.42 | 0.35 | 0.23 | 0.44 | 0.48 |
| **V+C** | 0.23 | 0.11 | 0.28 | 0.20 | 0.32 | 3.57 |
| **D+T** | 0.22 | 0.27 | 0.41 | 0.38 | 0.49 | 1.58 |
| | CD69 (MFI) | | | | | |
| | **Mock T only** | **CAR-T only** | **Mock T + T2** | **Mock T + A375M** | **CAR T + T2** | **CAR T + A375M** |
| **no inhibitor** | 0.00 | 0.41 | 0.59 | 0.08 | 0.93 | 9.41 |
| **DMSO** | 0.00 | 0.38 | 0.42 | 0.15 | 0.91 | 20.66 |
| **Vem** | 0.00 | 0.34 | 0.07 | 0.09 | 0.44 | 1.58 |
| **Dabra** | 0.03 | 0.51 | 0.31 | 0.07 | 0.66 | 5.62 |
| **Cobi** | 0.00 | 0.38 | 0.12 | 0.03 | 0.40 | 1.09 |
| **Tram** | 0.00 | 0.45 | 0.07 | 0.11 | 0.35 | 1.42 |
| **V+C** | 0.00 | 0.15 | 0.10 | 0.09 | 0.23 | 2.32 |
| **D+T** | 0.00 | 0.25 | 0.65 | 0.13 | 0.66 | 6.96 |

[1] MFI was determined by flow cytometry. See legend to figure 2 and materials and methods section for details.

**Table S3.** *p*-values of CD25 upregulation (paired students *t*-test) [1].

| Inhibitor | CAR-T Cells on T2 vs. CAR-T Cells on A375M | CAR-T Cells on A375M DMSO vs. Inhibitor[2] | CAR-T Cells on A375M[3] |
|---|---|---|---|
| no inhibitor | ∗ | | ns |
| DMSO control | ∗ | | |
| Vem | 0.07 | ∗ | ∗ |
| Dabra | ∗∗ | ns | |
| Cobi | ∗∗ | ns | ns |
| Tram | ∗∗∗ | 0.06 | |
| Vem & Cobi | 0.07 | ns | ns |
| Dabra & Tram | ∗∗ | ns | |

[1] The table depicts the results of statistical analyses related to Figure 2a. Relative expression of CD25 on CAR-transfected T cells after stimulation was compared. p-values were calculated using the paired Student's t test: (p>0.1: ns, p≤0.05: ∗, p≤0.01: ∗∗, p≤0.001: ∗∗∗; p-values >0.05 but ≤0.1 are indicated as numbers). [2] Comparison between the indicated inhibitor and the DMSO solvent control. [3] Comparison between each 2 corresponding inhibitors i.e. no inhibitor vs DMSO control, Vem vs Dabra, Cobi vs. Tram, and Vem & Cobi vs. Dabra & Tram.

**Table S4.** *p*-values of CD69 upregulation (paired students *t*-test) [1].

| Inhibitor | CAR-T Cells on T2 vs CAR-T Cells on A375M | CAR-T Cells on A375M DMSO vs. Inhibitor[2] | CAR-T Cells on A375M[3] |
|---|---|---|---|
| no inhibitor | 0.09 | | ns |
| DMSO control | ns | | |
| Vem | ∗ | ns | ns |
| Dabra | 0.10 | ns | |
| Cobi | ∗∗ | ns | ns |
| Tram | ∗ | ns | |
| Vem & Cobi | 0.07 | ns | ns |
| Dabra & Tram | ns | ns | |

[1] The table depicts the results of statistical analyses related to Figure 2b. Relative expression of CD69 on CAR-transfected T cells after stimulation was compared. p-values were calculated using the paired Student's t test: (p>0.1: ns, p≤0.05: ∗, p≤0.01: ∗∗; p-values >0.05 but ≤0.1 are indicated as numbers). [2] Comparison between the indicated inhibitor and the DMSO solvent control. [3] Comparison between each 2 corresponding inhibitors i.e. no inhibitor vs DMSO control, Vem vs Dabra, Cobi vs. Tram, and Vem & Cobi vs. Dabra & Tram.

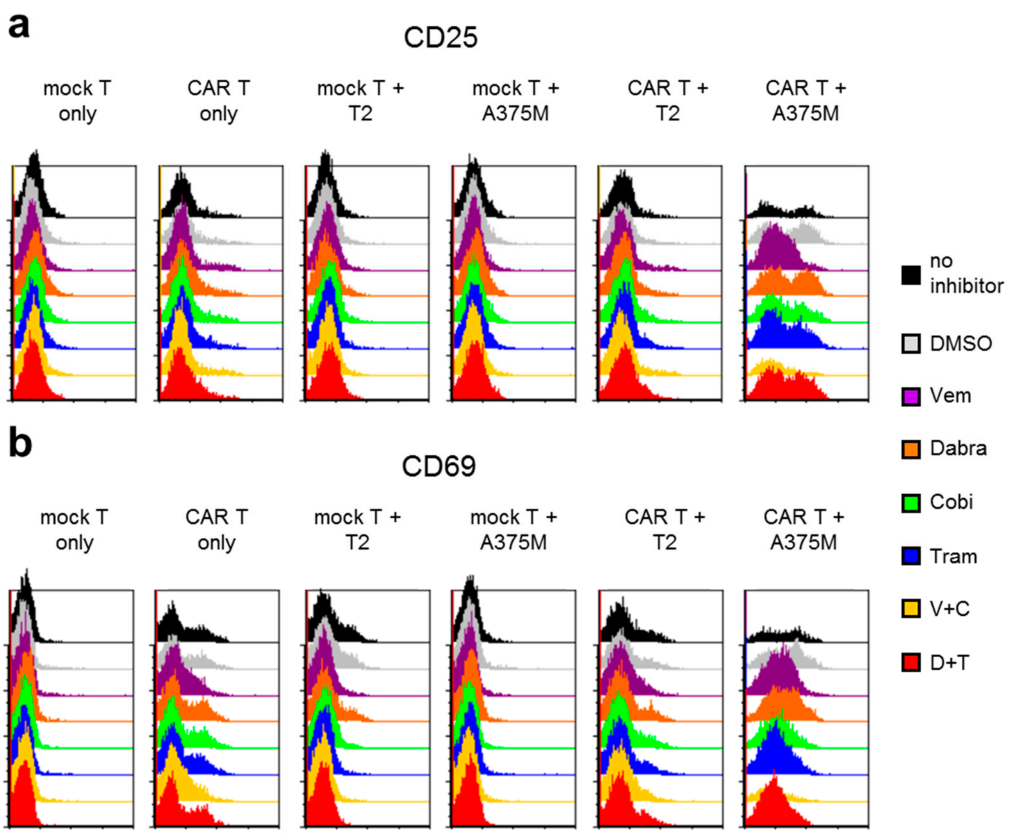


**Figure S1.** BRAF and MEK inhibitor treatment affects CAR-T-cell activation after antigen-specific stimulation. CAR-T cells were generated as described in figure 1. Four hours after electroporation, these cells were co-incubated over-night with CSPG4-negative T2 cells and the $CSPG4^{+}$ melanoma cell line A375M at a 1:1 ratio. Mock-transfected T cells were used as control. Co-incubations were performed in the absence of inhibitors (no inhibitor), in the presence of DMSO only (solvent control), or in the presence of the different kinase inhibitors, either alone or in combination. The used kinase inhibitors vemurafenib (Vem, V), dabrafenib (Dabra, D), cobimetinib (Cobi, C), and trametinib (Tram, T) were used in final concentrations listed in Table 1. Mock-transfected T cells stimulated with T2 or A375M, and mock-transfected T cells and CAR-T cells incubated without target cells served as negative controls. After 16 hours of co-incubation, the cells were harvested and stained for the activation markers CD25 (**a**) and CD69 (**b**) and measured by flow cytometry. Histograms of a representative donor are depicted.

**Table S5.** Original data used for Figure 3 [1].

| HD1 | | | | |
|---|---|---|---|---|
| **IL-2** | **TNF** | **IFNgamma** | | |
| pg/ml | pg/ml | pg/ml | | |
| 0 | 8 | 3 | | **T2 only** |
| 0 | 1 | 4 | | **A375M only** |
| 33 | 192 | 166 | | **Mock T + T2** |
| 1 | 14 | 20 | | **Mock T + A375M** |
| 28 | 353 | 454 | | **CAR T + T2** |
| 654 | 2303 | 7594 | **no inhibitor** | **CAR T + A375M** |
| 1 | 9 | 12 | | **T2 only** |
| 0 | 2 | 8 | | **A375M only** |
| 57 | 232 | 278 | | **Mock T + T2** |
| 1 | 9 | 23 | | **Mock T + A375M** |
| 18 | 421 | 397 | | **CAR T + T2** |
| 837 | 3234 | 9679 | **DMSO** | **CAR T + A375M** |
| 1 | 7 | 5 | | **T2 only** |
| 1 | 2 | 7 | | **A375M only** |
| 1 | 58 | 32 | | **Mock T + T2** |
| 1 | 4 | 13 | | **Mock T + A375M** |
| 7 | 224 | 407 | | **CAR T + T2** |
| 213 | 1257 | 2865 | **Vem** | **CAR T + A375M** |
| 1 | 4 | 5 | | **T2 only** |
| 1 | 2 | 10 | | **A375M only** |
| 16 | 149 | 332 | | **Mock T + T2** |
| 1 | 4 | 21 | | **Mock T + A375M** |
| 13 | 297 | 649 | | **CAR T + T2** |
| 674 | 2632 | 9460 | **Dabra** | **CAR T + A375M** |
| 1 | 1 | 6 | | **T2 only** |
| 0 | 0 | 9 | | **A375M only** |
| 10 | 18 | 65 | | **Mock T + T2** |
| 1 | 3 | 6 | | **Mock T + A375M** |
| 3 | 34 | 117 | | **CAR T + T2** |
| 125 | 562 | 3333 | **Cobi** | **CAR T + A375M** |
| 1 | 2 | 7 | | **T2 only** |
| 0 | 2 | 6 | | **A375M only** |
| 15 | 27 | 65 | | **Mock T + T2** |
| 1 | 3 | 8 | | **Mock T + A375M** |
| 3 | 52 | 129 | | **CAR T + T2** |
| 238 | 850 | 4026 | **Tram** | **CAR T + A375M** |
| 1 | 4 | 8 | | **T2 only** |
| 1 | 1 | 3 | | **A375M only** |
| 0 | 3 | 11 | **V+C** | **Mock T + T2** |

| | | | | |
|---|---|---|---|---|
| 0 | 0 | 5 | | **Mock T + A375M** |
| 2 | 26 | 179 | | **CAR T + T2** |
| 27 | 194 | 601 | | **CAR T + A375M** |
| 1 | 2 | 6 | | **T2 only** |
| 1 | 1 | 5 | | **A375M only** |
| 1 | 13 | 72 | | **Mock T + T2** |
| 1 | 2 | 9 | | **Mock T + A375M** |
| 2 | 33 | 128 | | **CAR T + T2** |
| 114 | 544 | 3217 | **D+T** | **CAR T + A375M** |
| HD2 | | | | |
| **IL-2** | **TNF** | **IFNgamma** | | |
| pg/ml | pg/ml | pg/ml | | |
| 0 | 3 | -1 | | **T2 only** |
| 0 | 0 | 0 | | **A375M only** |
| 29 | 76 | 324 | | **Mock T + T2** |
| 2 | 6 | 28 | | **Mock T + A375M** |
| 24 | 346 | 2614 | | **CAR T + T2** |
| 2238 | 5148 | 14468 | **no inhibitor** | **CAR T + A375M** |
| 0 | 6 | -2 | | **T2 only** |
| 0 | -1 | 0 | | **A375M only** |
| 14 | 95 | 306 | | **Mock T + T2** |
| 2 | 5 | 23 | | **Mock T + A375M** |
| 13 | 373 | 2133 | | **CAR T + T2** |
| 1975 | 5042 | 13321 | **DMSO** | **CAR T + A375M** |
| 0 | 4 | -1 | | **T2 only** |
| 0 | -1 | 0 | | **A375M only** |
| 0 | 9 | 4 | | **Mock T + T2** |
| 0 | 0 | 3 | | **Mock T + A375M** |
| 2 | 102 | 286 | | **CAR T + T2** |
| 154 | 1321 | 1416 | **Vem** | **CAR T + A375M** |
| 0 | 3 | 1 | | **T2 only** |
| 0 | 0 | 1 | | **A375M only** |
| 4 | 40 | 530 | | **Mock T + T2** |
| 0 | 1 | 9 | | **Mock T + A375M** |
| 5 | 127 | 2344 | | **CAR T + T2** |
| 1662 | 3986 | 14834 | **Dabra** | **CAR T + A375M** |
| 0 | 0 | 0 | | **T2 only** |
| -1 | 0 | 0 | | **A375M only** |
| 3 | 6 | 118 | | **Mock T + T2** |
| 0 | 0 | 6 | | **Mock T + A375M** |
| 2 | 21 | 396 | | **CAR T + T2** |
| 466 | 942 | 5575 | **Cobi** | **CAR T + A375M** |
| 0 | 0 | 2 | **Tram** | **T2 only** |

| | | | | |
|---|---|---|---|---|
| 0 | -1 | 2 | | **A375M only** |
| 4 | 10 | 108 | | **Mock T + T2** |
| 0 | -1 | 5 | | **Mock T + A375M** |
| 1 | 31 | 407 | | **CAR T + T2** |
| 801 | 1241 | 6632 | | **CAR T + A375M** |
| 0 | -1 | -2 | | **T2 only** |
| 0 | -1 | 1 | | **A375M only** |
| 0 | 2 | 10 | | **Mock T + T2** |
| 0 | 1 | 1 | | **Mock T + A375M** |
| 0 | 14 | 132 | | **CAR T + T2** |
| 485 | 649 | 3549 | **V+C** | **CAR T + A375M** |
| 0 | 0 | 1 | | **T2 only** |
| 0 | -1 | 0 | | **A375M only** |
| 0 | 3 | 106 | | **Mock T + T2** |
| 0 | 0 | -1 | | **Mock T + A375M** |
| 0 | 13 | 426 | | **CAR T + T2** |
| 690 | 1088 | 6763 | **D+T** | **CAR T + A375M** |

| HD3 | | | | |
|---|---|---|---|---|
| **IL-2** | **TNF** | **IFNgamma** | | |
| pg/ml | pg/ml | pg/ml | | |
| 0 | 1 | -1 | | **T2 only** |
| 0 | 1 | 0 | | **A375M only** |
| 69 | 16 | 95 | | **Mock T + T2** |
| 4 | 100 | 29 | | **Mock T + A375M** |
| 151 | 174 | 1432 | | **CAR T + T2** |
| 2100 | 2328 | 9225 | **no inhibitor** | **CAR T + A375M** |
| 0 | 11 | 0 | | **T2 only** |
| 0 | 1 | -1 | | **A375M only** |
| 53 | 59 | 195 | | **Mock T + T2** |
| 4 | 101 | 31 | | **Mock T + A375M** |
| 100 | 530 | 2580 | | **CAR T + T2** |
| 1848 | 2160 | 9508 | **DMSO** | **CAR T + A375M** |
| 0 | 4 | -1 | | **T2 only** |
| 0 | -1 | -1 | | **A375M only** |
| 1 | 13 | 3 | | **Mock T + T2** |
| 0 | 40 | 8 | | **Mock T + A375M** |
| 6 | 84 | 405 | | **CAR T + T2** |
| 384 | 841 | 2156 | **Vem** | **CAR T + A375M** |

| | | | | |
|---|---|---|---|---|
| | | | | |
| 0 | 1 | -1 | | **T2 only** |
| 0 | 1 | 0 | | **A375M only** |
| 33 | 11 | 178 | | **Mock T + T2** |
| 1 | 40 | 15 | | **Mock T + A375M** |
| 38 | 49 | 1473 | | **CAR T + T2** |
| 1473 | 1446 | 9365 | **Dabra** | **CAR T + A375M** |
| | | | | |
| 0 | 2 | -1 | | **T2 only** |
| 0 | 0 | 1 | | **A375M only** |
| 20 | 4 | 48 | | **Mock T + T2** |
| 0 | 4 | 1 | | **Mock T + A375M** |
| 18 | 14 | 266 | | **CAR T + T2** |
| 417 | 183 | 3021 | **Cobi** | **CAR T + A375M** |
| | | | | |
| 0 | 0 | -2 | | **T2 only** |
| 0 | 1 | 0 | | **A375M only** |
| 39 | 6 | 57 | | **Mock T + T2** |
| 2 | 22 | 15 | | **Mock T + A375M** |
| 51 | 35 | 498 | | **CAR T + T2** |
| 998 | 745 | 5199 | **Tram** | **CAR T + A375M** |
| | | | | |
| 1 | 1 | -1 | | **T2 only** |
| 1 | 0 | 1 | | **A375M only** |
| 1 | 1 | -1 | | **Mock T + T2** |
| 0 | 3 | 2 | | **Mock T + A375M** |
| 1 | 11 | 81 | | **CAR T + T2** |
| 120 | 110 | 695 | **V+C** | **CAR T + A375M** |
| | | | | |
| 0 | 1 | 0 | | **T2 only** |
| 0 | 0 | 0 | | **A375M only** |
| 5 | 3 | 60 | | **Mock T + T2** |
| 1 | 6 | 4 | | **Mock T + A375M** |
| 10 | 11 | 381 | | **CAR T + T2** |
| 680 | 358 | 3883 | **D+T** | **CAR T + A375M** |
| HD4 | | | | |
| **IL-2** | **TNF** | **IFNgamma** | | |
| pg/ml | pg/ml | pg/ml | | |
| | | | **no inhibitor** | |

| | | | | |
|---|---|---|---|---|
| 0 | 3 | -4 | | **T2 only** |
| 0 | 0 | -6 | | **A375M only** |
| 35 | 43 | 142 | | **Mock T + T2** |
| 1 | 8 | 21 | | **Mock T + A375M** |
| 30 | 124 | 1226 | | **CAR T + T2** |
| 2138 | 3159 | 9489 | | **CAR T + A375M** |
| | | | | |
| | | | | |
| 0 | 3 | -4 | | **T2 only** |
| 0 | 0 | -1 | | **A375M only** |
| 21 | 35 | 125 | | **Mock T + T2** |
| 1 | 8 | 23 | | **Mock T + A375M** |
| 21 | 113 | 1038 | | **CAR T + T2** |
| 2370 | 3405 | 9788 | **DMSO** | **CAR T + A375M** |
| | | | | |
| | | | | |
| 0 | 0 | -3 | | **T2 only** |
| 0 | 2 | 0 | | **A375M only** |
| 0 | 2 | -2 | | **Mock T + T2** |
| 0 | 1 | -1 | | **Mock T + A375M** |
| 3 | 31 | 280 | | **CAR T + T2** |
| 108 | 421 | 690 | **Vem** | **CAR T + A375M** |
| | | | | |
| | | | | |
| 0 | 0 | 1 | | **T2 only** |
| 0 | -1 | -3 | | **A375M only** |
| 13 | 18 | 329 | | **Mock T + T2** |
| 0 | 1 | 8 | | **Mock T + A375M** |
| 18 | 50 | 1653 | | **CAR T + T2** |
| 2038 | 2267 | 11235 | **Dabra** | **CAR T + A375M** |
| | | | | |
| | | | | |
| 0 | 0 | -4 | | **T2 only** |
| 0 | 1 | -1 | | **A375M only** |
| 6 | 3 | 97 | | **Mock T + T2** |
| 0 | 0 | 7 | | **Mock T + A375M** |
| 2 | 12 | 240 | | **CAR T + T2** |
| 660 | 283 | 3934 | **Cobi** | **CAR T + A375M** |
| | | | | |
| | | | | |
| 0 | 1 | -2 | | **T2 only** |
| 0 | 0 | 0 | | **A375M only** |
| 10 | 10 | 169 | | **Mock T + T2** |
| 0 | 2 | 5 | **Tram** | **Mock T +** |

| | | | | |
|---|---|---|---|---|
| | | | | **A375M** |
| 2 | 20 | 386 | | **CAR T + T2** |
| 1126 | 720 | 5828 | | **CAR T + A375M** |
| | | | | |
| | | | | |
| 0 | 1 | -4 | | **T2 only** |
| 0 | 0 | -3 | | **A375M only** |
| 2 | 1 | 51 | | **Mock T + T2** |
| 0 | 0 | 8 | | **Mock T + A375M** |
| 1 | 10 | 182 | | **CAR T + T2** |
| 794 | 266 | 3442 | **V+C** | **CAR T + A375M** |
| 0 | -1 | -4 | | |
| 0 | 58 | 85 | | |
| 0 | 1 | -6 | | **T2 only** |
| 0 | -1 | -2 | | **A375M only** |
| 27 | 17 | 625 | | **Mock T + T2** |
| 0 | 3 | 25 | | **Mock T + A375M** |
| 4 | 36 | 1157 | | **CAR T + T2** |
| 2012 | 2304 | 10720 | **D+T** | **CAR T + A375M** |
| n=4 | | | | |
| **IL-2** | **TNF** | **IFNgamma** | | |
| pg/ml | pg/ml | pg/ml | | |
| | | | | |
| | | | | |
| 0 | 4 | -1 | | **T2 only** |
| 0 | 0 | 0 | | **A375M only** |
| 42 | 82 | 182 | | **Mock T + T2** |
| 2 | 32 | 24 | | **Mock T + A375M** |
| 58 | 249 | 1432 | | **CAR T + T2** |
| 1782 | 3234 | 10194 | **no inhibitor** | **CAR T + A375M** |
| | | | | |
| | | | | |
| 0 | 7 | 1 | | **T2 only** |
| 0 | 0 | 1 | | **A375M only** |
| 36 | 105 | 226 | | **Mock T + T2** |
| 2 | 31 | 25 | | **Mock T + A375M** |
| 38 | 359 | 1537 | | **CAR T + T2** |
| 1758 | 3460 | 10574 | **DMSO** | **CAR T + A375M** |
| | | | | |
| | | | | |
| 0 | 4 | 0 | | **T2 only** |
| 0 | 0 | 1 | | **A375M only** |
| 1 | 20 | 9 | | **Mock T + T2** |
| 0 | 11 | 6 | **Vem** | **Mock T +** |

| | | | | |
|---|---|---|---|---|
| | | | | **A375M** |
| 4 | 110 | 344 | | **CAR T + T2** |
| 214 | 960 | 1782 | | **CAR T + A375M** |
| | | | | |
| | | | | |
| 0 | 2 | 2 | | **T2 only** |
| 0 | 1 | 2 | | **A375M only** |
| 16 | 55 | 342 | | **Mock T + T2** |
| 1 | 11 | 13 | | **Mock T + A375M** |
| 18 | 131 | 1530 | | **CAR T + T2** |
| 1462 | 2583 | 11224 | **Dabra** | **CAR T + A375M** |
| | | | | |
| | | | | |
| 0 | 1 | 0 | | **T2 only** |
| 0 | 0 | 2 | | **A375M only** |
| 10 | 8 | 82 | | **Mock T + T2** |
| 0 | 2 | 5 | | **Mock T + A375M** |
| 6 | 20 | 255 | | **CAR T + T2** |
| 417 | 493 | 3966 | **Cobi** | **CAR T + A375M** |
| | | | | |
| | | | | |
| 0 | 1 | 1 | | **T2 only** |
| 0 | 1 | 2 | | **A375M only** |
| 17 | 13 | 100 | | **Mock T + T2** |
| 1 | 6 | 8 | | **Mock T + A375M** |
| 14 | 35 | 355 | | **CAR T + T2** |
| 791 | 889 | 5421 | **Tram** | **CAR T + A375M** |
| | | | | |
| | | | | |
| 0 | 1 | 0 | | **T2 only** |
| 0 | 0 | 0 | | **A375M only** |
| 1 | 2 | 18 | | **Mock T + T2** |
| 0 | 1 | 4 | | **Mock T + A375M** |
| 1 | 15 | 144 | | **CAR T + T2** |
| 357 | 305 | 2072 | **V+C** | **CAR T + A375M** |
| | | | | |
| | | | | |
| 0 | 1 | 0 | | **T2 only** |
| 0 | 0 | 1 | | **A375M only** |
| 8 | 9 | 215 | | **Mock T + T2** |
| 0 | 3 | 9 | | **Mock T + A375M** |
| 4 | 23 | 523 | | **CAR T + T2** |
| 874 | 1073 | 6146 | **D+T** | **CAR T + A375M** |

[1] Cytokine concentrations were determined by Cytometric Bead Arrays. See legend to figure 3 and materials and methods section for details.

**Table S6.** *p*-values of IL-2 secretion (paired students *t*-test) [1].

| inhibitor | CAR-T cells on T2 vs CAR-T cells on A375M | CAR-T cells on A375M DMSO vs inhibitor[2] | CAR-T cells on A375M[3] |
| --- | --- | --- | --- |
| no inhibitor | * | | ns |
| DMSO control | * | | |
| Vem | * | * | * |
| Dabra | * | ** | |
| Cobi | * | ** | * |
| Tram | * | ** | |
| Vem & Cobi | ns | ** | ns |
| Dabra & Tram | ns | * | |

[1] The table depicts the results of statistical analyses related to Figure 3a. Secretion of IL-2 by CAR-transfected T cells after stimulation was compared. p-values were calculated using the paired Student's t test: ($p>0.1$: ns, $p\leq0.05$: *, $p\leq0.01$: **).[2] Comparison between the indicated inhibitor and the DMSO solvent control. [3] Comparison between each 2 corresponding inhibitors i.e. no inhibitor vs DMSO control, Vem vs Dabra, Cobi vs. Tram, and Vem & Cobi vs. Dabra & Tram.

**Table S7.** *p*-values of TNF secretion (paired students *t*-test) [1].

| inhibitor | CAR-T cells on T2 vs CAR-T cells on A375M | CAR-T cells on A375M DMSO vs inhibitor[2] | CAR-T cells on A375M[3] |
| --- | --- | --- | --- |
| no inhibitor | * | | ns |
| DMSO control | * | | |
| Vem | * | * | * |
| Dabra | * | ** | |
| Cobi | 0.07 | ** | ** |
| Tram | ** | * | |
| Vem & Cobi | 0.09 | ** | ns |
| Dabra & Tram | 0.09 | * | |

[1] The table depicts the results of statistical analyses related to Figure 3b. Secretion of TNF by CAR-transfected T cells after stimulation was compared. p-values were calculated using the paired Student's t test: ($p>0.1$: ns, $p\leq0.05$: *, $p\leq0.01$: **; p-values >0.05 but ≤0.1 are indicated as numbers). [2] Comparison between the indicated inhibitor and the DMSO solvent control. [3] Comparison between each 2 corresponding inhibitors i.e. no inhibitor vs DMSO control, Vem vs Dabra, Cobi vs. Tram, and Vem & Cobi vs. Dabra & Tram.

**Table S8.** *p*-values of IFNγ secretion (paired students *t*-test) [1].

| inhibitor | CAR-T cells on T2 vs CAR-T cells on A375M | CAR-T cells on A375M DMSO vs inhibitor[2] | CAR-T cells on A375M[3] |
|---|---|---|---|
| no inhibitor | ∗∗ | | ns |
| DMSO control | ∗∗ | | |
| Vem | ∗ | ∗∗ | ∗∗ |
| Dabra | ∗∗ | ns | |
| Cobi | ∗∗ | ∗∗∗ | ∗ |
| Tram | ∗∗ | ∗∗ | |
| Vem & Cobi | 0.10 | ∗∗ | ∗ |
| Dabra & Tram | ∗ | 0.09 | |

[1] The table depicts the results of statistical analyses related to Figure 3c. Secretion of IFNγ by CAR-transfected T cells after stimulation was compared. p-values were calculated using the paired Student's t test: ($p>0.1$: ns, $p\leq0.05$: ∗, $p\leq0.01$: ∗∗, $p\leq0.001$: ∗∗∗; p-values >0.05 but ≤0.1 are indicated as numbers). [2] Comparison between the indicated inhibitor and the DMSO solvent control. [3] Comparison between each 2 corresponding inhibitors i.e. no inhibitor vs DMSO control, Vem vs Dabra, Cobi vs. Tram, and Vem & Cobi vs. Dabra & Tram.

**Table S9.** Original data used for Figure 4 [1].

| HD1 | | | |
|---|---|---|---|
| **IL-6** | **IL-10** | | |
| pg/ml | pg/ml | | |
| | | | |
| | | | |
| 1 | 13 | | **T2 only** |
| 1 | 3 | | **A375M only** |
| 1 | 11 | | **Mock T + T2** |
| 3 | 3 | | **Mock T + A375M** |
| 2 | 13 | | **CAR T + T2** |
| 13 | 7 | **no inhibitor** | **CAR T + A375M** |
| | | | |
| | | | |
| 1 | 12 | | **T2 only** |
| 0 | 3 | | **A375M only** |
| 2 | 9 | | **Mock T + T2** |
| 1 | 2 | | **Mock T + A375M** |
| 1 | 10 | | **CAR T + T2** |
| 16 | 7 | **DMSO** | **CAR T + A375M** |
| | | | |
| | | | |
| 1 | 5 | | **T2 only** |
| 1 | 0 | | **A375M only** |
| 1 | 6 | | **Mock T + T2** |
| 1 | 0 | | **Mock T + A375M** |
| 1 | 6 | | **CAR T + T2** |
| 4 | 1 | **Vem** | **CAR T + A375M** |
| | | | |
| | | **Dabra** | |

| | | | |
|---|---|---|---|
| 1 | 11 | | **T2 only** |
| 1 | 1 | | **A375M only** |
| 7 | 11 | | **Mock T + T2** |
| 1 | 1 | | **Mock T + A375M** |
| 6 | 11 | | **CAR T + T2** |
| 538 | 4 | | **CAR T + A375M** |
| | | | |
| | | | |
| 0 | 4 | | **T2 only** |
| 0 | 0 | | **A375M only** |
| 1 | 4 | | **Mock T + T2** |
| 1 | 1 | | **Mock T + A375M** |
| 1 | 4 | | **CAR T + T2** |
| 63 | 1 | **Cobi** | **CAR T + A375M** |
| | | | |
| | | | |
| 1 | 4 | | **T2 only** |
| 1 | 1 | | **A375M only** |
| 0 | 5 | | **Mock T + T2** |
| 1 | 1 | | **Mock T + A375M** |
| 1 | 4 | | **CAR T + T2** |
| 72 | 1 | **Tram** | **CAR T + A375M** |
| | | | |
| | | | |
| 1 | 3 | | **T2 only** |
| 0 | 1 | | **A375M only** |
| 0 | 3 | | **Mock T + T2** |
| 0 | 0 | | **Mock T + A375M** |
| 0 | 3 | | **CAR T + T2** |
| 2 | 1 | **V+C** | **CAR T + A375M** |
| | | | |
| | | | |
| 1 | 4 | | **T2 only** |
| 1 | 0 | | **A375M only** |
| 1 | 4 | | **Mock T + T2** |
| 1 | 1 | | **Mock T + A375M** |
| 1 | 4 | | **CAR T + T2** |
| 231 | 2 | **D+T** | **CAR T + A375M** |
| HD2 | | | |
| **IL-6** | **IL-10** | | |
| pg/ml | pg/ml | | |
| | | | |
| | | | |
| 0 | 18 | | **T2 only** |
| 0 | 3 | | **A375M only** |
| 1 | 21 | | **Mock T + T2** |
| 0 | 3 | | **Mock T + A375M** |
| 4 | 20 | | **CAR T + T2** |
| 30 | 40 | **no inhibitor** | **CAR T + A375M** |
| | | | |
| | | | |
| 1 | 23 | **DMSO** | **T2 only** |

| | | | |
|---|---|---|---|
| 0 | 2 | | **A375M only** |
| 1 | 17 | | **Mock T + T2** |
| 0 | 2 | | **Mock T + A375M** |
| 4 | 16 | | **CAR T + T2** |
| 28 | 29 | | **CAR T + A375M** |
| | | | |
| | | | |
| 0 | 5 | | **T2 only** |
| -1 | 0 | | **A375M only** |
| 0 | 6 | | **Mock T + T2** |
| 0 | 1 | | **Mock T + A375M** |
| 1 | 8 | | **CAR T + T2** |
| 3 | 1 | **Vem** | **CAR T + A375M** |
| | | | |
| | | | |
| 3 | 20 | | **T2 only** |
| 0 | 1 | | **A375M only** |
| 5 | 19 | | **Mock T + T2** |
| 1 | 0 | | **Mock T + A375M** |
| 9 | 16 | | **CAR T + T2** |
| 903 | 14 | **Dabra** | **CAR T + A375M** |
| | | | |
| | | | |
| 1 | 7 | | **T2 only** |
| 0 | 0 | | **A375M only** |
| 0 | 5 | | **Mock T + T2** |
| 0 | 0 | | **Mock T + A375M** |
| 2 | 6 | | **CAR T + T2** |
| 204 | 4 | **Cobi** | **CAR T + A375M** |
| | | | |
| | | | |
| 0 | 10 | | **T2 only** |
| 0 | 0 | | **A375M only** |
| 0 | 7 | | **Mock T + T2** |
| 0 | 0 | | **Mock T + A375M** |
| 1 | 8 | | **CAR T + T2** |
| 141 | 5 | **Tram** | **CAR T + A375M** |
| | | | |
| | | | |
| 0 | 4 | | **T2 only** |
| -1 | 0 | | **A375M only** |
| 0 | 3 | | **Mock T + T2** |
| -1 | 0 | | **Mock T + A375M** |
| 1 | 4 | | **CAR T + T2** |
| 9 | 1 | **V+C** | **CAR T + A375M** |
| | | | |
| | | | |
| 0 | 6 | | **T2 only** |
| 0 | 1 | | **A375M only** |
| 1 | 4 | | **Mock T + T2** |
| 0 | 1 | | **Mock T + A375M** |
| 3 | 6 | **D+T** | **CAR T + T2** |

| | | | |
|---|---|---|---|
| 618 | 3 | | **CAR T + A375M** |
| HD3 | | | |
| **IL-6** | **IL-10** | | |
| pg/ml | pg/ml | | |
| | | | |
| | | | |
| 0 | 23 | | **T2 only** |
| 1 | 3 | | **A375M only** |
| 1 | 19 | | **Mock T + T2** |
| 2 | 3 | | **Mock T + A375M** |
| 2 | 22 | | **CAR T + T2** |
| 19 | 27 | **no inhibitor** | **CAR T + A375M** |
| | | | |
| | | | |
| 1 | 21 | | **T2 only** |
| 0 | 2 | | **A375M only** |
| 1 | 20 | | **Mock T + T2** |
| 1 | 2 | | **Mock T + A375M** |
| 1 | 19 | | **CAR T + T2** |
| 16 | 21 | **DMSO** | **CAR T + A375M** |
| | | | |
| | | | |
| 0 | 5 | | **T2 only** |
| 1 | 0 | | **A375M only** |
| 0 | 6 | | **Mock T + T2** |
| 0 | 1 | | **Mock T + A375M** |
| 1 | 7 | | **CAR T + T2** |
| 3 | 2 | **Vem** | **CAR T + A375M** |
| | | | |
| | | | |
| 1 | 20 | | **T2 only** |
| 1 | 0 | | **A375M only** |
| 4 | 17 | | **Mock T + T2** |
| 16 | 1 | | **Mock T + A375M** |
| 3 | 17 | | **CAR T + T2** |
| 1052 | 8 | **Dabra** | **CAR T + A375M** |
| | | | |
| | | | |
| 0 | 5 | | **T2 only** |
| 1 | 1 | | **A375M only** |
| 0 | 5 | | **Mock T + T2** |
| 1 | 1 | | **Mock T + A375M** |
| 1 | 5 | | **CAR T + T2** |
| 79 | 2 | **Cobi** | **CAR T + A375M** |
| | | | |
| | | | |
| 1 | 10 | | **T2 only** |
| 1 | 2 | | **A375M only** |
| 1 | 9 | | **Mock T + T2** |
| 0 | 1 | | **Mock T + A375M** |
| 1 | 10 | | **CAR T + T2** |
| 90 | 7 | **Tram** | **CAR T + A375M** |
| | | [illegible] | |

| | | | |
|---|---|---|---|
| | | | |
| 1 | 4 | | **T2 only** |
| 1 | 1 | | **A375M only** |
| 0 | 3 | | **Mock T + T2** |
| 1 | 0 | | **Mock T + A375M** |
| 1 | 3 | | **CAR T + T2** |
| 2 | 0 | | **CAR T + A375M** |
| | | | |
| | | | |
| 0 | 5 | | **T2 only** |
| 1 | 1 | | **A375M only** |
| 1 | 5 | | **Mock T + T2** |
| 2 | 1 | | **Mock T + A375M** |
| 2 | 5 | | **CAR T + T2** |
| 547 | 2 | **D+T** | **CAR T + A375M** |
| HD4 | | | |
| **IL-6** | **IL-10** | | |
| pg/ml | pg/ml | | |
| | | | |
| | | | |
| 0 | 2 | | **T2 only** |
| 0 | 1 | | **A375M only** |
| 1 | 1 | | **Mock T + T2** |
| 0 | 0 | | **Mock T + A375M** |
| 1 | 3 | | **CAR T + T2** |
| 58 | 70 | **no inhibitor** | **CAR T + A375M** |
| | | | |
| | | | |
| 0 | 2 | | **T2 only** |
| 1 | 0 | | **A375M only** |
| 1 | 1 | | **Mock T + T2** |
| 0 | 0 | | **Mock T + A375M** |
| 0 | 3 | | **CAR T + T2** |
| 62 | 79 | **DMSO** | **CAR T + A375M** |
| | | | |
| | | | |
| 0 | 1 | | **T2 only** |
| 1 | 1 | | **A375M only** |
| 1 | 1 | | **Mock T + T2** |
| 0 | 0 | | **Mock T + A375M** |
| 0 | 1 | | **CAR T + T2** |
| 1 | 0 | **Vem** | **CAR T + A375M** |
| | | | |
| | | | |
| 0 | 2 | | **T2 only** |
| 1 | 0 | | **A375M only** |
| 6 | 1 | | **Mock T + T2** |
| 1 | 0 | | **Mock T + A375M** |
| 1 | 2 | | **CAR T + T2** |
| 2895 | 39 | **Dabra** | **CAR T + A375M** |
| | | | |
| | | | |
| 0 | 1 | | **T2 only** |
| 1 | 0 | **Cobi** | **A375M only** |

| | | | |
|---|---|---|---|
| 0 | 1 | | **Mock T + T2** |
| 0 | 0 | | **Mock T + A375M** |
| 1 | 1 | | **CAR T + T2** |
| 469 | 1 | | **CAR T + A375M** |
| | | | |
| | | | |
| 0 | 1 | | **T2 only** |
| 0 | 1 | | **A375M only** |
| 1 | 1 | | **Mock T + T2** |
| 0 | 0 | | **Mock T + A375M** |
| 0 | 1 | | **CAR T + T2** |
| 497 | 5 | **Tram** | **CAR T + A375M** |
| | | | |
| | | | |
| 0 | 1 | | **T2 only** |
| -1 | 0 | | **A375M only** |
| 0 | 1 | | **Mock T + T2** |
| 1 | 0 | | **Mock T + A375M** |
| 1 | 1 | | **CAR T + T2** |
| 21 | 0 | **V+C** | **CAR T + A375M** |
| 0 | 0 | | |
| 0 | 0 | | |
| 0 | 1 | | **T2 only** |
| 1 | 0 | | **A375M only** |
| 5 | 1 | | **Mock T + T2** |
| 2 | 0 | | **Mock T + A375M** |
| 1 | 1 | | **CAR T + T2** |
| 2128 | 30 | **D+T** | **CAR T + A375M** |
| n=4 | | | |
| **IL-6** | **IL-10** | | |
| pg/ml | pg/ml | | |
| | | | |
| | | | |
| 0 | 14 | | **T2 only** |
| 1 | 2 | | **A375M only** |
| 1 | 13 | | **Mock T + T2** |
| 1 | 2 | | **Mock T + A375M** |
| 2 | 14 | | **CAR T + T2** |
| 30 | 36 | **no inhibitor** | **CAR T + A375M** |
| | | | |
| | | | |
| 1 | 15 | | **T2 only** |
| 0 | 2 | | **A375M only** |
| 1 | 12 | | **Mock T + T2** |
| 1 | 2 | | **Mock T + A375M** |
| 2 | 12 | | **CAR T + T2** |
| 30 | 34 | **DMSO** | **CAR T + A375M** |
| | | | |
| | | | |
| 0 | 4 | | **T2 only** |
| 1 | 1 | **Vem** | **A375M only** |

| | | | |
|---|---|---|---|
| 0 | 4 | | **Mock T + T2** |
| 0 | 0 | | **Mock T + A375M** |
| 1 | 6 | | **CAR T + T2** |
| 3 | 1 | | **CAR T + A375M** |
| | | | |
| | | | |
| 1 | 13 | | **T2 only** |
| 1 | 1 | | **A375M only** |
| 5 | 12 | | **Mock T + T2** |
| 5 | 0 | | **Mock T + A375M** |
| 5 | 12 | | **CAR T + T2** |
| 1347 | 16 | **Dabra** | **CAR T + A375M** |
| | | | |
| | | | |
| 0 | 4 | | **T2 only** |
| 1 | 1 | | **A375M only** |
| 0 | 4 | | **Mock T + T2** |
| 1 | 1 | | **Mock T + A375M** |
| 1 | 4 | | **CAR T + T2** |
| 204 | 2 | **Cobi** | **CAR T + A375M** |
| | | | |
| | | | |
| 0 | 6 | | **T2 only** |
| 0 | 1 | | **A375M only** |
| 1 | 5 | | **Mock T + T2** |
| 0 | 1 | | **Mock T + A375M** |
| 1 | 6 | | **CAR T + T2** |
| 200 | 5 | **Tram** | **CAR T + A375M** |
| | | | |
| | | | |
| 0 | 3 | | **T2 only** |
| 0 | 0 | | **A375M only** |
| 0 | 3 | | **Mock T + T2** |
| 0 | 0 | | **Mock T + A375M** |
| 0 | 3 | | **CAR T + T2** |
| 8 | 1 | **V+C** | **CAR T + A375M** |
| | | | |
| | | | |
| 0 | 4 | | **T2 only** |
| 1 | 0 | | **A375M only** |
| 2 | 4 | | **Mock T + T2** |
| 1 | 1 | | **Mock T + A375M** |
| 2 | 4 | | **CAR T + T2** |
| 881 | 10 | **D+T** | **CAR T + A375M** |

[1]Cytokine concentrations were determined by Cytometric Bead Arrays. See legend to figure 3 and materials and methods section for details.

**Table S10.** *p*-values of IL-6 secretion (paired students *t*-test) [1].

| inhibitor | CAR-T cells on T2 vs CAR-T cells on A375M | CAR-T cells on A375M DMSO vs inhibitor[2] | CAR-T cells on A375M[3] |
|---|---|---|---|
| no inhibitor | 0.073 | | ns |
| DMSO control | 0.081 | | |
| Vem | * | 0.09 | 0.08 |
| Dabra | 0.08 | 0.08 | |
| Cobi | ns | ns | ns |
| Tram | ns | ns | |
| Vem & Cobi | ns | * | ns |
| Dabra & Tram | ns | ns | |

[1] The table depicts the results of statistical analyses related to Figure 4a. Secretion of IL-6 by CAR-transfected T cells after stimulation was compared. p-values were calculated using the paired Student's t test: ($p>0.1$: ns, $p\leq0.05$: *; p-values >0.05 but ≤0.1 are indicated as numbers). [2] Comparison between the indicated inhibitor and the DMSO solvent control. [3] Comparison between each 2 corresponding inhibitors i.e. no inhibitor vs DMSO control, Vem vs Dabra, Cobi vs. Tram, and Vem & Cobi vs. Dabra & Tram.

**Table S11.** *p*-values of IL-10 secretion (paired students *t*-test) [1].

| inhibitor | CAR-T cells on T2 vs CAR-T cells on A375M | CAR-T cells on A375M DMSO vs inhibitor[2] | CAR-T cells on A375M[3] |
|---|---|---|---|
| no inhibitor | ns | | ns |
| DMSO control | ns | | |
| Vem | * | ns | ns |
| Dabra | ns | ns | |
| Cobi | 0.07 | ns | ns |
| Tram | ns | ns | |
| Vem & Cobi | * | ns | ns |
| Dabra & Tram | ns | 0.07 | |

[1] The table depicts the results of statistical analyses related to Figure 4b. Secretion of IL-10 by CAR-transfected T cells after stimulation was compared. p-values were calculated using the paired Student's t test: ($p>0.1$: ns, $p\leq0.05$: *, $p\leq0.01$; p-values >0.05 but ≤0.1 are indicated as numbers). [2] Comparison between the indicated inhibitor and the DMSO solvent control. [3] Comparison between each 2 corresponding inhibitors i.e. no inhibitor vs DMSO control, Vem vs Dabra, Cobi vs. Tram, and Vem & Cobi vs. Dabra & Tram.

**Table S12.** Original data used for figure 5 [1].

| **HD 1** | | | | |
|---|---|---|---|---|
| **relative lysis** | | | | |
| | | | | |
| | T2 | 54:1 | 18:1 | 6:1 |
| no inhibitor | Mock T | 1.45 | -1.04 | 0.21 |

| | | | | |
|---|---|---|---|---|
| DMSO | | 0.93 | -2.59 | -0.10 |
| Vem | | -0.21 | 0.21 | -0.62 |
| Dabra | | -1.14 | 0.31 | -1.14 |
| Cobi | | -0.10 | -0.72 | -0.47 |
| Tram | | 0.10 | 0.31 | 0.10 |
| V+C | | -1.76 | 0.47 | 0.10 |
| D+T | | -1.14 | -0.72 | 1.35 |
| | | | | |
| | | | | |
| | A375M | 54:1 | 18:1 | 6:1 |
| no inhibitor | Mock T | -2.56 | -2.31 | -2.25 |
| DMSO | | -1.85 | -2.31 | -3.17 |
| Vem | | -2.34 | -2.50 | -2.50 |
| Dabra | | -1.73 | -1.85 | -2.37 |
| Cobi | | -1.60 | -2.07 | -2.31 |
| Tram | | -2.40 | -1.51 | -3.27 |
| V+C | | -2.82 | -2.96 | -2.34 |
| D+T | | -2.64 | -2.22 | -2.43 |
| | | | | |
| | | | | |
| | T2 | 54:1 | 18:1 | 6:1 |
| no inhibitor | CAR T | -0.88 | -1.81 | -0.62 |
| DMSO | | -0.31 | -2.54 | -1.24 |
| Vem | | -2.33 | -2.33 | -1.45 |
| Dabra | | 0.62 | -1.60 | -2.74 |
| Cobi | | -1.92 | -2.12 | -1.14 |
| Tram | | -1.19 | -2.28 | -2.17 |
| V+C | | -0.78 | -1.24 | -2.80 |
| D+T | | -1.92 | -2.69 | -2.07 |
| | | | | |
| | | | | |
| | A375M | 54:1 | 18:1 | 6:1 |
| no inhibitor | CAR T | 7.55 | 4.07 | 2.16 |
| DMSO | | 8.60 | 4.96 | 3.76 |
| Vem | | 9.12 | 6.50 | 2.87 |
| Dabra | | 9.12 | 7.34 | 3.21 |
| Cobi | | 3.61 | 1.08 | -0.34 |
| Tram | | 9.40 | 6.47 | 2.28 |
| V+C | | 4.28 | 1.48 | -0.34 |
| D+T | | 7.40 | 4.35 | 1.11 |
| **HD 2** | | | | |
| **relative lysis** | | | | |
| | | | | |
| | T2 | 54:1 | 18:1 | 6:1 |
| no inhibitor | Mock T | 0.26 | -1.39 | 0.69 |
| DMSO | | -1.65 | -2.82 | 3.03 |
| Vem | | -2.69 | -2.69 | -0.74 |
| Dabra | | -1.56 | 0.17 | 0.56 |
| Cobi | | -0.35 | -1.91 | -2.56 |
| Tram | | -2.08 | -1.78 | 3.94 |
| V+C | | -0.35 | -2.95 | 2.64 |

| | | | | |
|---|---|---|---|---|
| D+T | | -1.52 | -2.69 | 1.73 |
| | | | | |
| | | | | |
| | A375M | 54:1 | 18:1 | 6:1 |
| no inhibitor | Mock T | -0.76 | -0.35 | -1.06 |
| DMSO | | 0.40 | 0.76 | 0.45 |
| Vem | | 0.30 | 0.10 | -0.86 |
| Dabra | | 0.71 | 0.20 | -0.76 |
| Cobi | | -0.45 | -0.05 | -0.91 |
| Tram | | -1.06 | -0.81 | -0.50 |
| V+C | | -0.86 | 0.00 | -1.01 |
| D+T | | 0.61 | -1.51 | -0.96 |
| | | | | |
| | | | | |
| | T2 | 54:1 | 18:1 | 6:1 |
| no inhibitor | CAR T | 0.26 | -1.56 | 0.56 |
| DMSO | | -1.30 | -1.91 | -0.87 |
| Vem | | 0.09 | -1.21 | -1.00 |
| Dabra | | -0.78 | -0.61 | -2.17 |
| Cobi | | -2.08 | -1.65 | -2.17 |
| Tram | | -0.43 | -0.69 | 0.17 |
| V+C | | -2.77 | -1.99 | -2.69 |
| D+T | | -1.47 | -1.91 | -2.17 |
| | | | | |
| | | | | |
| | A375M | 54:1 | 18:1 | 6:1 |
| no inhibitor | CAR T | 7.82 | 3.18 | 0.71 |
| DMSO | | 7.52 | 2.88 | 0.81 |
| Vem | | 7.02 | 3.43 | -1.31 |
| Dabra | | 8.58 | 3.84 | -0.15 |
| Cobi | | 2.78 | 0.86 | -1.21 |
| Tram | | 8.13 | 2.68 | -0.86 |
| V+C | | 2.37 | -0.71 | -1.72 |
| D+T | | 6.51 | 1.16 | -0.96 |
| **HD 3** | | | | |
| **relative lysis** | | | | |
| | | | | |
| | T2 | 54:1 | 18:1 | 6:1 |
| no inhibitor | Mock T | -0.90 | -1.33 | -1.06 |
| DMSO | | 1.39 | -1.59 | -2.06 |
| Vem | | -1.06 | -2.06 | -2.72 |
| Dabra | | -0.73 | -1.79 | -1.79 |
| Cobi | | -2.92 | -2.46 | -3.32 |
| Tram | | -1.59 | -1.73 | -1.39 |
| V+C | | -1.79 | -2.19 | -2.72 |
| D+T | | -1.79 | -1.59 | -1.92 |
| | | | | |
| | | | | |
| | A375M | 54:1 | 18:1 | 6:1 |
| no inhibitor | Mock T | 5.59 | 5.93 | 4.30 |
| DMSO | | 2.59 | 4.22 | 0.11 |

| | | | | |
|---|---|---|---|---|
| Vem | | 1.31 | -1.18 | 1.82 |
| Dabra | | 2.08 | 1.31 | 0.19 |
| Cobi | | -0.32 | -2.12 | -0.15 |
| Tram | | 0.45 | 0.36 | 1.13 |
| V+C | | -2.29 | -2.80 | -0.92 |
| D+T | | 3.45 | 1.13 | 0.02 |
| | | | | |
| | | | | |
| | T2 | 54:1 | 18:1 | 6:1 |
| no inhibitor | CAR T | 6.47 | -1.69 | 1.10 |
| DMSO | | 1.79 | -0.40 | 3.78 |
| Vem | | 0.00 | -2.26 | -1.59 |
| Dabra | | -0.33 | -1.53 | -0.80 |
| Cobi | | -2.39 | -2.06 | -2.12 |
| Tram | | -1.92 | -2.12 | -2.32 |
| V+C | | -2.06 | -2.26 | -2.46 |
| D+T | | -0.93 | -1.59 | -2.46 |
| | | | | |
| | | | | |
| | A375M | 54:1 | 18:1 | 6:1 |
| no inhibitor | CAR T | 25.11 | 12.18 | 6.27 |
| DMSO | | 23.56 | 12.52 | 5.59 |
| Vem | | 15.26 | 5.24 | 2.68 |
| Dabra | | 26.30 | 13.89 | 7.47 |
| Cobi | | 14.92 | 4.39 | 2.68 |
| Tram | | 18.00 | 7.73 | 4.39 |
| V+C | | 8.00 | 0.28 | -2.38 |
| D+T | | 24.10 | 7.13 | 3.02 |
| **n=3** | | | | |
| **relative lysis** | | | | |
| | | | | |
| | T2 | 54:1 | 18:1 | 6:1 |
| no inhibitor | Mock T | 0.27 | -1.25 | -0.05 |
| DMSO | | 0.23 | -2.33 | 0.29 |
| Vem | | -1.32 | -1.51 | -1.36 |
| Dabra | | -1.14 | -0.44 | -0.79 |
| Cobi | | -1.12 | -1.70 | -2.11 |
| Tram | | -1.19 | -1.06 | 0.88 |
| V+C | | -1.30 | -1.56 | 0.01 |
| D+T | | -1.48 | -1.67 | 0.38 |
| | | | | |
| | | | | |
| | A375M | 54:1 | 18:1 | 6:1 |
| no inhibitor | Mock T | 0.76 | 1.09 | 0.33 |
| DMSO | | 0.38 | 0.89 | -0.87 |
| Vem | | -0.24 | -1.19 | -0.51 |
| Dabra | | 0.35 | -0.11 | -0.98 |
| Cobi | | -0.79 | -1.41 | -1.12 |
| Tram | | -1.00 | -0.65 | -0.88 |
| V+C | | -1.99 | -1.92 | -1.42 |
| D+T | | 0.47 | -0.87 | -1.12 |

| | | | | |
|---|---|---|---|---|
| | | | | |
| | | | | |
| | T2 | 54:1 | 18:1 | 6:1 |
| no inhibitor | CAR T | 1.95 | -1.69 | 0.35 |
| DMSO | | 0.06 | -1.61 | 0.56 |
| Vem | | -0.75 | -1.93 | -1.35 |
| Dabra | | -0.16 | -1.25 | -1.90 |
| Cobi | | -2.13 | -1.94 | -1.81 |
| Tram | | -1.18 | -1.70 | -1.44 |
| V+C | | -1.87 | -1.83 | -2.65 |
| D+T | | -1.44 | -2.06 | -2.23 |
| | | | | |
| | | | | |
| | A375M | 54:1 | 18:1 | 6:1 |
| no inhibitor | CAR T | 13.49 | 6.48 | 3.05 |
| DMSO | | 13.23 | 6.79 | 3.38 |
| Vem | | 10.47 | 5.06 | 1.41 |
| Dabra | | 14.67 | 8.35 | 3.51 |
| Cobi | | 7.10 | 2.11 | 0.37 |
| Tram | | 11.84 | 5.62 | 1.94 |
| V+C | | 4.89 | 0.35 | -1.48 |
| D+T | | 12.67 | 4.21 | 1.06 |

[1]Cytotoxicity was determined in a $Cr^{51}$-release assay. See legend to figure 4 and materials and methods section for details.

**Table S13.** *p*-values of cytotixicity (paired students *t*-test) [1].

| | CAR-T cells on T2 vs CAR-T cells on A375M | | CAR-T cells on A375M DMSO vs inhibitor[2] | | CAR-T cells on A375M[3] | |
|---|---|---|---|---|---|---|
| inhibitor | 54 : 1[4] | 18 : 1[4] | 54 : 1[4] | 18 : 1[4] | 54 : 1[4] | 18 : 1[4] |
| no inhibitor | * | * | | | ns | ns |
| DMSO control | * | * | | | | |
| Vem | ** | ** | ns | ns | ns | ns |
| Dabra | * | * | 0.06 | * | | |
| Cobi | * | * | ** | * | ** | * |
| Tram | ** | * | ns | ns | | |
| Vem & Cobi | * | ** | * | 0.05 | 0.08 | * |
| Dabra & Tram | * | * | ns | 0.09 | | |

[1] The table depicts the results of statistical analyses related to Figure 5. Lytic activity of CAR-transfected T cells against T2 and A375M was compared. p-values were calculated using the paired Student’s t test: ($p>0.1$: ns, $p \leq 0.05$: *, $p \leq 0.01$: **; p-values $>0.05$ but $\leq 0.1$ are indicated as numbers). [2] Comparison between the indicated inhibitor and the DMSO solvent control. [3] Comparison between each 2 corresponding inhibitors i.e. no inhibitor vs DMSO control, Vem vs Dabra, Cobi vs. Tram, and Vem & Cobi vs. Dabra & Tram. [4] Effector to target ratio.
